# Thermodynamic Factors Controlling Electron Transfer among the Terminal Electron Acceptors of Photosystem I: Insights from Kinetic Modelling

**DOI:** 10.3390/ijms25189795

**Published:** 2024-09-10

**Authors:** Stefano Santabarbara, Anna Paola Casazza

**Affiliations:** Photosynthesis Research Unit, Consiglio Nazionale delle Ricerche, Via A. Corti 12, 20133 Milano, Italy; annapaola.casazza@ibba.cnr.it

**Keywords:** electron transfer, standard Gibbs free energy difference, reorganisation energy, tunnelling barrier, iron–sulphur cluster, (phyllo)quinone

## Abstract

Photosystem I is a key component of primary energy conversion in oxygenic photosynthesis. Electron transfer reactions in Photosystem I take place across two parallel electron transfer chains that converge after a few electron transfer steps, sharing both the terminal electron acceptors, which are a series of three iron–sulphur (Fe-S) clusters known as $F_{X}$, $F_{A}$, and $F_{B}$, and the terminal donor, $P_{700}$. The two electron transfer chains show kinetic differences which are, due to their close geometrical symmetry, mainly attributable to the tuning of the physicochemical reactivity of the bound cofactors, exerted by the protein surroundings. The factors controlling the rate of electron transfer between the terminal Fe-S clusters are still not fully understood due to the difficulties of monitoring these events directly. Here we present a discussion concerning the driving forces associated with electron transfer between $F_{X}$ and $F_{A}$ as well as between $F_{A}$ and $F_{B}$, employing a tunnelling-based description of the reaction rates coupled with the kinetic modelling of forward and recombination reactions. It is concluded that the reorganisation energy for $F_{X}^{-}$ oxidation shall be lower than 1 eV. Moreover, it is suggested that the analysis of mutants with altered $F_{A}$ redox properties can also provide useful information concerning the upstream phylloquinone cofactor energetics.

## 1. Introduction

Photosystem I (PSI) is a fundamental component of the oxygenic photosynthetic electron transport chain. It catalyses the light-dependent oxidation of soluble electron shuttles, most commonly either the copper-binding protein plastocyanin or the small cytochrome *c*_6_, at its so-called donor side and the reduction of ferredoxin, which is also a soluble protein, at its acceptor side. Photosystem I is a very large membrane-embedded cofactor-protein supercomplex, composed of more than 10 protein subunits (e.g., refs. [1,2]), the exact number of which depends on the species. From an operational perspective, it can be considered as being composed, analogously to the other photosystems, of two functional moieties. The first is the core complex, which has the role of performing primary charge separation and successive charge stabilisation through electron transfer (ET) reactions, involving a series of redox-active cofactors. The core complex also harbours cofactors that have a light-harvesting function and are, as those composing the ET chain, well conserved throughout evolution. The second functional unit is the peripheral antenna system, which has a light-harvesting function only. Its composition, in terms of proteins and chromophores, varies significantly among different organisms.

**Figure 1:**
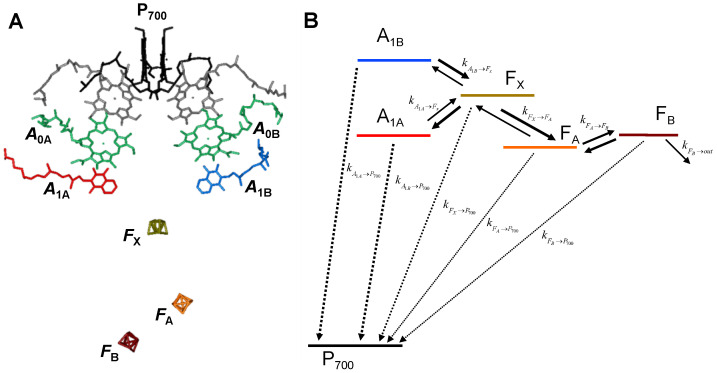
(**A**) Arrangement of the redox-active cofactors in PS I (from the structural model of Jordan et al. [3]). The terminal electron donor ($P_{700}^{\left(+\right)}$) is considered as a Chl *a*/Chl *a*’ hetero-dimer and is shown in black; the so-called accessory Chl *a* are unlabeled and are shown in grey; the primary Chl *a* electron acceptors $A_{0A,B}^{\left(-\right)}$ are shown in green; the phylloquinone $A_{1A}^{\left(-\right)}$ is own in red and $A_{1B}^{\left(-\right)}$ in blue. The terminal 4Fe-4S clusters, $F_{X}$, $F_{A}$, and $F_{B}$ are shown in gold, orange, and burgundy colour, respectively. (**B**) Kinetic scheme used in the simulations of electron transfer reactions. The solid arrow indicates forward and backward rates between pairs of successive redox-active cofactors. Dashed and dotted lines represent charge recombination reactions between individual cofactors and $P_{700}^{+}$, involving the phylloquinones and iron–sulphur clusters, respectively. The colour code is the same as in (**A**) and is maintained throughout the manuscript. Note that the energy gaps are not scaled. Properly scaled $E^{0}$ are shown in Figure 2 and Figure 3.

The characteristics of the subunits composing the core complex of PSI appear to be generally well-conserved throughout evolutionary divergent species, particularly considering the two largest subunits, PsaA and PsaB, which form a heterodimer and coordinate, together with the core-antenna pigments, the majority of the redox-active cofactors. Also well-conserved is the PsaC subunit, which binds two 4Fe-4S clusters, referred to as $F_{A}$ and $F_{B}$, acting as the terminal electron acceptors within the photosystem and being, in turn, responsible for ferredoxin reduction. Structural studies [3,4,5] have shown that, in analogy to all other known photosynthetic reaction centres, the PsaA- and PsaB-coordinated cofactors, identified as participating in ET reactions, are organised in a highly symmetric fashion. The redox-active cofactors display a mirror symmetry arrangement with respect to the reference axis, which is putatively perpendicular to the membrane plane (Figure 1A). The symmetric organisation of the cofactors results in the presence of two putative ET chains, which are, however, not completely independent, sharing the (hetero)dimer of Chlorophyll (Chl) *a*/Chl *a*’ (the latter being an epimer [3]), assigned to the long-lived terminal electron donor $P_{700}^{\left(+\right)}$ and the 4Fe-4S cluster $F_{X}$, which is the last PsaA/PsaB-bound acceptor before the PsaC-bound $F_{A}$ and $F_{B}$ centres. Both $P_{700}$, where the number indicates the maximal absorption bleaching upon oxidation [2,6], and $F_{X}$ are coordinated at the interface of PsaA and PsaB.

A general consensus has been reached over the years that in PSI, the two parallel cofactor chains are both functional ([2,7,8,9,10,11,12] and references therein). This represents a major functional difference from Photosystem II and the purple bacteria Reaction Centre (RC), in which primary photochemistry and ET reactions involve exclusively one cofactor chain. In these type of photosystems, the terminal electron acceptor, the quinone $Q_{B}$, is structurally part of the “inactive” chain, but accepts electrons from the “photochemically functional chain” bound quinone, $Q_{A}$. The need to control inter-quinone ET and the full reduction of $Q_{B}$ to quinole by two consecutive charge separation events is thought to be one of the main reasons for having an asymmetric, also referred to as mono-directional, ET in Type II photosynthetic RC. In PSI, and generally in Type I RC to whom it belongs, ET reactions involve only one-electron exchange, so the need to control the accumulation of either oxidative or reducing equivalents at any level of the ET chain is not stringent.

Nonetheless, the probability with which electrons are transferred through each ET chain of PSI remains somewhat debated, with figures ranging from almost equal utilisation [12,13,14,15,16,17,18,19] to very asymmetric proportions in favour of the PsaA-coordinated branch [20,21,22,23,24]. Further functional differences are also apparent at the level of intermediate steps, particularly in the lifetime of oxidation of the reduced phylloquinone, $A_{1}$, by the next acceptor in the chain, $F_{X}$. This reaction shows a complex kinetic behaviour, which in its simplest description is accounted by two main phases characterised by lifetimes of 5–20 ns and 200–300 ns [6,7,8,9,10,25,26]. Studies of mutants at the level of the phylloquinone binding site have led to the assignment of the 200–300 ns phase to the oxidation of $A_{1A}^{-}$ [6,7,8,9,10,27,28,29,30,31] (the subscript indicates the subunit which primarily coordinates the cofactor) and the 5–20 ns phase to the oxidation of $A_{1B}^{-}$ [6,7,8,9,10,27,31]. These phases also show significantly different temperature dependences, with the slowest one displaying a larger activation energy (65–130 meV) compared to the fast one (6–20 meV) [32,33,34]. Since $F_{X}$ is a common cofactor to both reactions, and because of the similarity in the co-ordination geometry of the phylloquinones, the one-order-of-magnitude difference in the $A_{1A}^{-}$ and $A_{1B}^{-}$ oxidation kinetics was attributed within the framework of the ET theory (e.g., refs. [35,36,37] and references therein) to an asymmetry in the driving force for these reactions, with $A_{1B}^{-}$ oxidation being more thermodynamically favourable (e.g., $\Delta G_{A_{1B}\to F_{X}}^{0}$ < $\Delta G_{A_{1A}\to F_{X}}^{0}$) [7,11,38,39]. Even though there is a general consensus concerning this interpretation, different estimates for the free energies have been advanced, also depending on the approach utilised for their evaluation ([12] and reference therein). Because of the very negative value of the phylloquinone potential, direct redox titration is, at best, cumbersome [40]. Contradictory results were also obtained for the titration of $F_{X}$, although its potential ($E_{F_{X}}^{0}$) can be safely regarded as less than −680 mV ([41,42,43] and see discussion in [6]). Moreover, even direct titration can suffer from the bias linked to the progressive accumulation of charges on the acceptor side (i.e., on $F_{A}$, $F_{B}$, $F_{X}$, and so on). The direct electrochemically determined midpoint potentials can therefore differ, also significantly, from the “operational” ones, when the nearby cofactors are oxidised instead (see Ptsushenko et al. [44] for further discussion). Thus, most of the estimated $A_{1A}$ and $A_{1B}$ redox potentials were derived either from the ET theory-based modelling of the oxidation kinetic [7,11,38,39] or from structure-based computational methods [44,45,46,47]. Nonetheless, it should be considered that both approaches require some specific simplifications and assumptions in order to address the problem. Hence, the estimations obtained from either of these methods contain some margin of uncertainty. A detailed description of these issues is beyond the scope of the present paper. However, currently, only two energetic models have been proven to account for the oxidation kinetics at room temperature, their temperature dependence [48] and the effect of mutations in the phylloquinone binding site [49]. It is worth noting that however, information is more abundant for mutants affecting the $A_{1A}^{-}$ to $F_{X}$ reaction than its counterpart,. These are the so-called “weak driving force” and “large driving force” scenarios. In the former, $A_{1A}^{-}$ oxidation is associated with a free energy difference of ~−30 meV < $\Delta G_{A_{1A}\to F_{X}}^{0}$< +30 meV whereas $A_{1B}^{-}$ oxidation is associated with $\Delta G_{A_{1B}\to F_{X}}^{0}$< −50 meV (note that the values are broad and rounded because they depend on the exact parameter set describing the reaction rate constants) [7,11,12,48,49]. It is worth noting that in the lower driving force limit, $A_{1A}^{-}$ oxidation becomes thermodynamically unfavourable. Within the “weak driving force” configuration, experimental observations are semi-quantitatively reproduced when considering a total reorganisation energy ($\lambda_{tot}$) associated with phylloquinone oxidation, as well as $F_{A}$ reduction by $F_{X}$, of about 0.7 eV [7,11,12,48,49]. The “large driving force” scenario invokes that both $\Delta G_{A_{1A}\to F_{X}}^{0}$ and $\Delta G_{A_{1B}\to F_{X}}^{0}$ < −50/75 meV and that $\Delta G_{A_{1B}\to F_{X}}^{0}$ << $\Delta G_{A_{1A}\to F_{X}}^{0}$, with the former corresponding to driving forces even larger than ~150 meV [39,44]. This energetic configuration, however, requires the consideration of much larger reorganisation energies, in the order of 1 eV [48,49], to describe the experimental phylloquinone oxidation kinetics. When assuming a homogenous value of $\lambda_{tot}$ for all the ET reactions, the difference in the values used to simulate $A_{1}^{-}$ oxidation (i.e., 1 eV with respect to 0.7 eV) results in pronounced differences in the simulations of $F_{X}$ oxidation for the two energetic configurations described above, even when keeping all other relevant simulation parameters, including $\Delta G_{F_{X}\to F_{A}}^{0}$, which is set at ~−150 meV [6,7], equal. In this framework, the electron transfer from $F_{X}$ to the terminal Fe-S clusters is predicted to be about 5-fold slower for the large $A_{1}^{-}$ oxidation driving force than for the weak $A_{1}^{-}$ oxidation driving force energetics ([48] and vide infra for further detail).

The redox potential of the iron–sulphur clusters $F_{A}$ an $F_{B}$ is, together with that of $P_{700}^{+}$, the most accurately determined, even in spite of some variability in the estimations [6,50,51,52,53]. Knowledge of these values will increase the margin of accuracy of kinetic modelling predictions. Nonetheless, the dynamics of ET involving these redox centres are not unambiguously understood yet. This is mainly due to the optical properties of the Fe-S cluster, which possess a broad, relatively unstructured spectrum that abundantly overlaps with those of other PSI-bound chromophores [54,55]. Moreover, ET involving species having almost identical absorption spectra implies a small differential extinction coefficient, making their direct detection by optical methods extremely challenging. Most of the kinetic information relies then on alternative methods, exploiting changes in the electric field through the protein milieu caused by the electron displacement along the ET chain. These include direct methods, such as photovoltage [56,57,58,59,60], and indirect optical probes, like the ET-dependent local-stark effect on chlorophylls and carotenoids [25]. From these methods, a relatively broad range of values for the $F_{X}$ to $F_{A}$/$F_{B}$ transfer time have been proposed, ranging from less than 25 ns to ~300 ns ([26,61] and references therein). A kinetic phase characterised by lifetimes of 160–180 ns, attributed to ET between the iron–sulphur clusters, was also resolved in mutants where the $A_{1A}^{-}$ oxidation phase, which otherwise overlaps with it, was slowed down more than three times [30,62,63]. A component with a similar lifetime is also resolvable in wild-type photosystems in temperature dependence studies, when it appears to have a lower activation energy, and becomes, therefore, discernible from the slow phase of $A_{1(A)}^{-}$ oxidation upon cooling [33,34]. Electron transfer between $F_{A}$ and $F_{B}$ is even less characterised, as the positioning of these cofactors leads to a vectorial ET almost parallel to the membrane plane (perpendicular to the symmetry axis), which is unfavourable for both local-Stark and photo-voltage detection. Hence, either only the collective transfer to $F_{A}$/$F_{B}$ is generally detected or, alternatively, transfer to $F_{A}$ alone after $F_{B}$ is chemically inactivated/destabilised [26,61]. It is commonly assumed that the transfer time across all of the Fe-S clusters shall be faster than 500 ns^−1^ μs to account for the rapid reduction of pre-bound ferredoxin, which takes place in about 1 to 3 μs, although even faster sub-microsecond kinetics have been reported [64].

In order to gain further insight into the dynamics of ET reactions involving the Fe-S of PSI, kinetic simulations are presented, in which the effect of the most crucial thermodynamic parameters, i.e., the standard free energy difference and the reorganisation energy, are explored. The ET reactions involving the Fe-S clusters are contextualised within the “weak” and “large” driving force energetic scenarios for $A_{1}^{-}$ oxidation. The simulations provide predictions that can in principle be tested experimentally, allowing for a better understanding of these ET events.

## 2. Results and Discussion

In order to gain some additional information concerning the ET reactions involving the 4Fe-4S centres in PSI, i.e., the reduction of $F_{A}$ by $F_{X}^{-}$ (hereafter the sign indicates the reduced state of the FeS centre, not its net charge) and the successive reduction of $F_{B}$ by $F_{A}^{-}$, kinetics simulations were performed starting from the two energetic scenarios, which provide a satisfactory, semi-quantitative description for the wild-type PSI. Subsequently, perturbations of parameters that control the rate constants of ET between the FeS clusters, mainly the standard free energies ($\Delta G_{DA}^{0}$) and the reorganisation energy ($\lambda_{tot}$), were introduced. A brief description of the choice of parameters that are common to all, or most, of the reactions included in the model is provided in the Materials and Methods section (Section 3), whereas reaction-specific values of parameters are discussed when introduced.

### 2.1. “Weak Driving Force” Scenario for $A_{1}^{-}$ Oxidation

**Figure 2:**
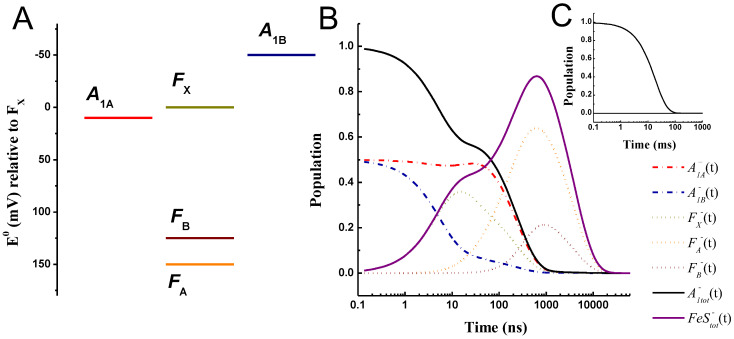
(**A**) Reference energetic scheme for the “weak driving force” scenario for $A_{1}^{-}$ oxidation; standard redox potentials ($E^{0}$) are shown relative to $F_{X}$. (**B**) Simulated population evolution of the individual redox-active cofactors. $A_{1A}^{-}(t)$: red dash-dotted line; $A_{1B}^{-}(t)$: blue dash-dotted line; $F_{X}^{-}(t)$: golden dotted line; $F_{A}^{-}(t)$: orange dotted line; $F_{B}^{-}(t)$: burgundy dotted line. Also shown are the total population evolutions of $A_{1,tot}^{-}(t)=A_{1A}^{-}(t)+A_{1B}^{-}(t)$ (black line) and $FeS_{tot}^{-}(t)=F_{X}^{-}(t)+F_{A}^{-}(t)+F_{B}^{-}(t)$ (purple line). The inset (**C**) shows the recombination kinetics simulated in the absence of an exit from the system ($k_{out}$ = 0). Temperature: 290 K.

Figure 2 shows the simulated population evolutions of the reduced ET cofactors in PSI, after the initial population of phylloquinones $A_{1A}$ and $A_{1B}$, within the so-called “weak driving force” framework. The parameters used in the calculations are reported in Table 1. In brief, the standard free energy difference for $A_{1A}^{-}$ and $A_{1B}^{-}$ oxidation were taken as $\Delta G_{A_{1A}\to F_{X}}^{0}$ = +10 meV and $\Delta G_{A_{1B}\to F_{X}}^{0}$ = −50 meV, and those for the 4Fe-4S centre oxidation were $\Delta G_{F_{X}\to F_{A}}^{0}$ = −150 meV and $\Delta G_{F_{A}\to F_{B}}^{0}$ = +25 meV. The latter two fall within the range derivable from direct redox titrations of the FeS cofactors [50,51,52,53]. Setting the midpoint potential of $F_{B}$ at −555 mV, it then results that $E_{F_{A}}^{0}$ = −530 mV, $E_{F_{X}}^{0}$ = −680 mV, $E_{A_{1A}}^{0}$ = −670 mV, and $E_{A_{1B}}^{0}$ = −730 mV. Because of the relative spread of experimentally retrieved $E^{0}$ values, and considering the effect of piling-up reducing equivalents upon titrations, which likely result in a bias in the determined FeS clusters redox potentials, the free energy differences calculated from the experimental titrations and those employed in the simulations might and shall not match exactly. Those used in the simulations rather reflect the so-called “operational” potentials. Even with these approximations, the general consideration that the energy gap between $F_{X}$ and $F_{A}$ is relatively large, whereas the one between $F_{A}$ and $F_{B}$ is shallow, with the two PsaC-coordinated centres being almost iso-potential, remains valid. For the parameters described above, and in general for the “weak driving force” model, it is possible to consider a common value of the reorganisation energy for the $A_{1A}^{-}$, $A_{1B}^{-}$, and $F_{X}^{-}$ oxidation equal to 0.7 eV, whereas a larger value of 0.9 eV was considered for $F_{A}^{-}$ oxidation. A value of $\lambda_{tot}$ = 0.7 eV corresponds to the indicative consensus obtained by Dutton and coworkers [37,65] from an analysis and survey of ET reactions in a broad range of protein systems. Using a larger value of $\lambda_{tot}$ for the $F_{A}\to F_{B}$ reaction can be justified on two lines of thought. The first is that the outer shell reconfiguration (the $\lambda_{o}$ component of the reorganisation energy) is expected to be larger for reactions involving metal centres only with respect to organic cofactors, and this shall in principle apply to the $F_{X}\to F_{A}$ reaction, too. The second and probably more significant factor is that the PsaC-bound $F_{A}$ and $F_{B}$ clusters are, on average, more exposed to the bulk water environment. This is particularly the case for $F_{B}$. Thus, the approximation that considers the cofactors embedded in a homogenous medium of low static permittivity ($\varepsilon_{s}$, as defined in Equation (2) in Section 3) ceases to be valid [66,67]. The partial exposure, or proximity, to bulk water, whose $\varepsilon_{s}$ value is much larger than that of proteins (80 vs. 2.5–3.5, e.g., refs. [66,67]), would then tend to increase $\lambda_{o}$ significantly (e.g., refs. [66,68]). This is then the dominant consideration for adopting a larger value of $\lambda_{tot}$ for the reactions involving the terminal iron–sulphur clusters, within the “weak driving force” scenario.

Specific frequencies of the mean coupled nuclear modes were considered for $A_{1}^{-}$ oxidation, corresponding to $\hbar {\bar{\omega }}_{A_{1A}\to F_{X}}$ = 22 meV (175 cm^−1^) and $\hbar {\bar{\omega }}_{A_{1B}\to F_{X}}$ = 46 meV (375 cm^−1^). These values were obtained from the analysis of the phylloquinone oxidation kinetics as a function of the temperature [69] and resulted in an improvement of the simulations of the temperature dependence of the kinetics when explicitly considered. A coupling $\hbar {\bar{\omega }}_{DA}$ = 34 meV (275 cm^−1^), which is the mean of the values employed for the quinone oxidation, was used for all other reactions. In the kinetic scheme shown above (Figure 1B), also the recombination reactions between all reduced cofactors and $P_{700}^{+}$ were included. In the simulations of the recombination reactions, $E_{P_{700}}^{0}$ was +450 mV, in accordance with direct estimations from redox titrations [6], and the reorganisation energy was 0.9 eV for all recombination reactions between any of the reduced cofactors and $P_{700}^{+}$. Adopting the same value of $\lambda_{tot}$ for these reactions can be justified, given that in the description of the outer sphere reorganisation, $\lambda_{o}$, there is a term that is inversely proportional to the donor–acceptor distance (Equation (2), e.g., refs. [35,36,67]). All other factors in Equation (2) being equal, $\lambda_{o}$ becomes smaller for shorter cofactor distances and tends to a constant value for large separations. This is the case of recombination reactions since the distance between $P_{700}^{+}$ and any of the reduced cofactors is always larger than 20 Å. To minimise the number of freely adjustable parameters in the simulations, the largest value of $\lambda_{tot}$ used for forward reactions was then also adopted for all recombination processes.

The recombination reactions have virtually no influence on the simulations of forward ET kinetics because these rates are several orders of magnitude slower than productive reactions. This is due to the large distances between the cofactor pairs, even in the presence of large favourable driving forces for all of these processes (see Section S1 of the Supplementary Materials). However, their inclusion allows us to compare the simulated and experimentally determined recombination kinetics, as shown in Figure 2C, by simply suppressing the exit rate from the system (i.e., $F_{B}^{-}$ oxidation by external electron acceptors).

For the parameters discussed above, it is obtained that, at room temperature (290 K), the system is characterised by five lifetimes (the number corresponding to the states present in the kinetics scheme) of 5.2, 22.5, 136, 243, and 3808 ns, which are common to the description of the population evolution of all cofactors. The 5.2, 22.5, and 243 ns lifetimes are those also retrieved from a three-state model that does not explicitly include $F_{A}$ and $F_{B}$ (e.g., refs. [7,11,12,48,49] and see Supplementary Materials Section S1, Figure S1 and Table S1) so that the remaining lifetimes of 136 and 3808 ns can be related to electron transfer reactions involving the terminal iron–sulphur cluster directly. The longest-lived lifetime corresponds to the exit from the system when this is considered ($F_{B}\to Out$). This reaction is not modelled according to the ET theory; a phenomenological rate of 1 × 10^−3^ ns^−1^ is considered instead, and it is set to zero when necessary to simulate the recombination reactions. Within this energetic scheme, the oxidation of $A_{1B}^{-}$ is dominated by the two fastest components (~5 and 22.5 ns), resulting in an average decay lifetime of 46 ns, whereas the oxidation of $A_{1A}^{-}$ is dominated by the 243 ns component, and the resulting average decay is 331 ns. Despite the assumption of equal initial population at time zero of $A_{1A}^{-}$ and $A_{1B}^{-}$, the predicted ratio of fast (5 ns and 22.5 ns) to slow (all remaining ones) oxidation phases is 0.33:0.67, which was interpreted as a transient inter-quinone population transfer [70]. Since the detail of the model predictions concerning the phylloquinone ET reactions has already been discussed previously (e.g., refs. [7,11,12,48,49]), more attention will be dedicated here to the transfer involving the 4Fe-4S clusters. Within the above-mentioned kinetic/energetic scheme, the average reduction time of $F_{X}$ is predicted to be relatively fast and described uniquely by the 5.2 ns component. In contrast, the successive oxidation is multiphasic, dominated by the 22.5 and 243 ns lifetimes, giving rise to an average decay lifetime ($\tau_{av}$) of 199 ns, which is only slightly slower than the value simulated for the total quinone oxidation ($A_{1,tot}^{-}$ = $A_{1A}^{-}$ + $A_{1B}^{-}$), being 160 ns. Similarly close values are obtained when comparing the mean population lifetime (i.e., the first moment of the population temporal evolution, $\tau_{m}$), which is estimated as being 900 ns for $F_{X}^{-}$ with respect to 805 ns for $A_{1,tot}^{-}$ oxidations. The $\tau_{m}$ values are larger than the $\tau_{av}$ values because of the increased weight of the slowest component in the parameter estimation, which is at least one order of magnitude slower than all other lifetimes. When the latter is omitted from the calculations, the mean lifetimes for $F_{X}^{-}$ and $A_{1,tot}^{-}$ become 240 and 243 ns, respectively, which are then close to $\tau_{av}$. Yet, irrespective of the parameters considered, these cofactors relax to their neutral state almost simultaneously. This would explain the difficulties in detecting an electrogenic phase specifically associated with the $F_{X}$ oxidation/reduction reactions, since this would broadly overlap kinetically with $A_{1}^{-}$ oxidation. The successive redox acceptors $F_{A}$ and $F_{B}$ are reduced with average lifetimes of 192 and 243 ns, respectively, that is, almost coordinately with the relaxation of $A_{1,tot}^{-}$ and $F_{X}^{-}$, and are oxidised with average lifetimes of 3.6 μs and 1.9 μs, respectively, clearly limited by the rate of exit from the system. The simulated mean lifetimes of $F_{A}^{-}$ and $F_{B}^{-}$ population evolutions are 4.0 μs and 4.2 μs, respectively, again indicating that the terminal acceptors are oxidised almost simultaneously. This is due to the weak, slightly endergonic driving force associated with $F_{A}^{-}$ oxidation, which leads to the partitioning of reducing equivalents in favour of this cofactor, with respect to the terminal acceptor $F_{B}$.

Figure 2C shows the predicted recombination in the absence of an exit from the system, where the oxidation of the terminal 4Fe-S cluster is dominated by a lifetime of 19 ms. This lifetime replaces the longest-lived ~4 μs lifetime predicted from the simulations in the presence of an acceptor. The simulated recombination lifetime falls well within the spread of values reported in the literature, which most commonly are in the range of 10–200 ms (see [6,26,71,72,73,74] and reference therein). It is also worth mentioning that the observed lifetime, however, does not reflect the (inverse) direct recombination rate from the terminal 4Fe-4S clusters, which are simulated as 2.1 × 10^−17^ ns^−1^ and 4.8 × 10^−22^ ns^−1^ for the reactions involving $F_{A}^{-}$ and $F_{B}^{-}$, respectively. Rather, the recombination rate/lifetime is determined by the energetically uphill repopulation of the upstream cofactors, chiefly $F_{X}^{-}$. The predicted recombination kinetics are virtually unaffected within the energetic framework discussed above by arbitrarily setting to zero the values of all recombination rates to $P_{700}^{+}$, excluding the direct recombination from the phylloquinones (Supplementary Materials Section S1, Figure S2).

### 2.2. “Large Driving Force” Scenario for $A_{1}^{-}$ Oxidation

**Figure 3:**
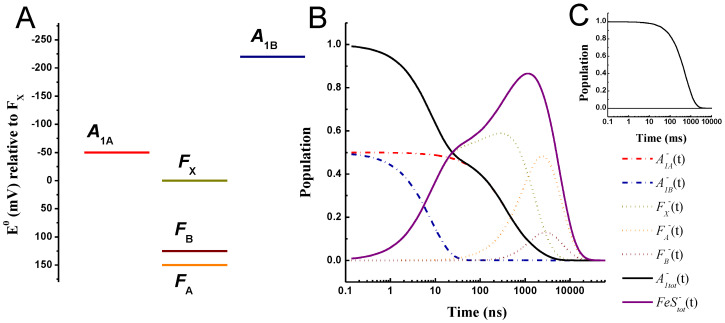
(**A**) Reference energetic scheme for the “large driving force” scenario for $A_{1}^{-}$ oxidation; standard redox potentials ($E^{0}$) are shown relative to $F_{X}$. (**B**) Simulated population evolution of the individual redox-active cofactors. $A_{1A}^{-}(t)$: red dash-dotted line; $A_{1B}^{-}(t)$: blue dash-dotted line, $F_{X}^{-}(t)$: golden dot golden line; $F_{A}^{-}(t)$: orange dotted line; $F_{B}^{-}(t)$: burgundy dotted line. Also shown are the total population evolutions of $A_{1,tot}^{-}(t)=A_{1A}^{-}(t)+A_{1B}^{-}(t)$ (black line) and $FeS_{tot}^{-}(t)=F_{X}^{-}(t)+F_{A}^{-}(t)+F_{B}^{-}(t)$ (purple line). The inset (**C**) shows the recombination kinetics simulated in the absence of an exit from the system ($k_{out}$ = 0). Temperature: 290 K.

Figure 3 shows the simulations obtained within the “large driving force” scenario for $A_{1}^{-}$ oxidation. In this case, we considered values of $\Delta G_{A_{1A}\to F_{X}}^{0}$ = −50 meV and $\Delta G_{A_{1B}\to F_{X}}^{0}$ = −220 meV as suggested by Milanovsky et al. [39], whereas the free energies for the other reactions were the same as discussed above, $\Delta G_{F_{X}\to F_{A}}^{0}$ = −150 meV and $\Delta G_{F_{A}\to F_{B}}^{0}$ = +25 meV. Setting the midpoint potential of $F_{B}$ at −555 mV, it then results that $E_{F_{A}}^{0}$ = −530 mV, $E_{F_{X}}^{0}$ = −680 mV, $E_{A_{1A}}^{0}$ = −730 mV, and $E_{A_{1B}}^{0}$ = −900 mV. The parameter set employed in the calculations is listed in Table 2. In order to facilitate the comparison between the two energetic scenarios considered, whenever feasible, the same parameters were adopted in the simulations. The main difference resides in the reorganisation energy value because, within the “large driving force” scheme, a value of $\lambda_{tot}$ = 0.7 eV associated with the quinone reactions resulted in simulated lifetimes that are too fast with respect to the measured ones, particularly for the slowest phase of $A_{1}^{-}$ oxidation. The slowest phase of $A_{1}^{-}$ oxidation would be simulated by a lifetime of ~100 ns, which is at least two times faster than the value retrieved from the experiments, i.e., ~250–360 ns (see Section S3 of the Supplementary Materials, Figures S4 and S5). It was therefore necessary, as also discussed previously [48], to use a larger value for $\lambda_{tot}$ of 1 eV for these ET steps, which was extended to the simulation of all other forward and recombination reactions. Within this energetic/kinetic scenario, the simulated lifetimes, common to all reactions, are 8.2, 295, 307, 1411, and 4415 ns. The 8.2, 307, and 1411 ns lifetimes are also retrieved for a kinetic scheme that does not explicitly consider the terminal iron–sulphur centres (Section S1, Figure S1 and Table S1 of the Supplementary Materials). Therefore, the remaining 295 and 4415 ns are associated mainly with ET processes directly involving $F_{A}$ and $F_{B}$.

The oxidation of $A_{1B}^{-}$ is dominated by the 8.2 ns lifetime and that of $A_{1A}^{-}$ by the 307 ns one, with some contribution from the 1.4 μs component. The fast–to–slow ratio of $A_{1,tot}^{-}$ oxidation phases is simulated as 0.5:0.5, corresponding to the initial populations of these cofactors. Different oxidation phase partitions could be straightforwardly simulated by setting the boundary conditions accordingly. This, in turn, implies an asymmetric charge separation/stabilisation between the two active ET chains of PSI (e.g., refs. [20,21,22,23,24]). Discussion of this issue is, however, beyond the scope of this work. For equal initial populations of $A_{1B}^{-}$ and $A_{1A}^{-}$, the resulting average lifetimes are 10.5 and 733 ns, respectively, and 371 ns for $A_{1,tot}^{-}$. The predicted oxidation of $A_{1A}^{-}$ is somewhat slower, within this energetic scheme and for the conditions described, due to the significant contribution of the 1.4 μs phase, whose exact value is in part linked to ET between the Fe-S clusters. Within this model, the reduction of $F_{X}$ is multiphasic, with contributions from all the sub-microsecond components, i.e., 8.2, 295, and 307 ns (Table 2), resulting in an average rise lifetime of 143 ns. In contrast, $F_{X}^{-}$ oxidation is largely dominated by the 1.4 μs phase, corresponding closely to the average oxidation ($\tau_{av}$) time and the mean lifetimes of the population evolution ($\tau_{m}$), both of which are 1.6 μs. This makes it slower compared to the 1.1 μs estimated for $A_{1,tot}^{-}$.

As apparent from the inspection of the population evolutions (Figure 3B), in this energetic scenario, the oxidation of $F_{X}^{-}$ clearly follows that of $A_{1,tot}^{-}$, whereas the two processes largely overlapped in the simulations performed within the small driving force energetic scheme (Figure 2B). This is associated with the slower rate of $F_{A}$ reduction from $F_{X}^{-}$ due to the larger reorganisation energy (1 eV rather than 0.7 eV). Although not necessarily impossible, it would be unusual that the reorganisation for an ET event between 4Fe-4S cluster was to be lower than the one associated with ET from phylloquinones to an iron–sulphur centre. For this reason, as well as to minimise the number of variable parameters, $\lambda_{tot}$ was set to the same value. Simulations for different values of the reorganisation energy are, however, explored in Section S4 of the Supplementary Materials where the effect of this parameter can be better appreciated (Figure S7).

The terminal electron acceptors $F_{A}$ and $F_{B}$ are reduced with average lifetimes of 1.2 μs and 808 ns, respectively, and oxidised with average lifetimes of 3.4 and 1.8 μs, respectively. The terminal acceptor oxidation is, basically, determined by the rate of exit from the system. The slower simulated dynamics of $F_{A}^{-}$ oxidation are, as in the “weak driving force” scenario, due to a slightly endergonic reduction of $F_{B}$. Nonetheless, the mean lifetimes for $F_{A}^{-}$ and $F_{B}^{-}$ are 5.8 and 6.2 μs, indicating that, as also discussed above, the two centres are predicted to be oxidised almost concertedly by diffusible acceptors.

When the rate of electron donation from $F_{B}^{-}$ is suppressed in order to simulate the recombination reactions, these are characterised by a lifetime of ~570 ms. This value falls rather outside the spread of values reported in the literature, where the slowest ones are in the order of 100–200 ms [6,26,71,72,73,74]. Yet, no specific effort has been made here to reach a good agreement between the measured and simulated recombination rates.

An improvement in the match between modelled (between 174 and 89 ms) and experimental values is obtained by increasing $\hbar {\bar{\omega }}_{DA}$ to 55 meV (450 cm^−1^) (Section S2, Figure S3 of the Supplementary Materials), which is the average recommended consensus value from a survey in redox-active proteins [37]. Variation of the values of $\lambda_{tot}$ (Section S3, Figure S6) or application of the parameter sets employed in the “weak driving force” model (Section S4, Figure S8), as discussed above, does not appear to improve the description of the recombination kinetics within the “large driving force” energetic scheme. Nonetheless, as in the “weak driving force” case, the recombination rate is limited by the uphill repopulation of $F_{X}^{-}$ and, especially, $A_{1(A,B)}^{-}$. As discussed by Cherepanov and coworkers [75], a more detailed description of the Franck–Condon function, involving multiple nuclear modes and specific electron–phonon coupling terms, as well as explicit consideration of $A_{0(A,B)}^{-}$ (the upstream electron donor), might be required to describe the recombination reactions in detail, at least within the “large driving force” energetic picture.

### 2.3. Effect of Changing the Driving Force of $F_{X}^{-}$ Oxidation ($\Delta G_{F_{X}\to F_{A}}^{0}$)

The most straightforward change in PSI energetics that affects the ET between the iron–sulphur cluster directly involves the tuning of the redox potential of the terminal electron acceptors $F_{A}$ and $F_{B}$. Mutations in the PsaC subunit that modify the coordination and hence the redox properties of $F_{A}$ and $F_{B}$ have already been reported [76,77,78,79,80,81,82,83,84,85,86,87]. However, the effect of these mutations on the $A_{1A,B}^{-}$ oxidation kinetics has not been investigated in detail, since the main target of these studies was to identify the specific nature and coordination site of the terminal acceptor, which has been a matter of controversy before being definitively elucidated by the availability of high-resolution structural models. Hence, these studies targeted primarily the residues directly involved in the binding of the FeS clusters [76,77,78,79,80,81,82,83,84,85,86,87], and the kinetic information reported related almost exclusively to the recombination rather than the forward ET reactions [76,77,78,79,80,81,82,83,84,85]. Similarly, mutants affecting either the direct coordination of $F_{X}$ [88,89,90,91] and, in some cases, nearby residues altering the cluster binding niche [91,92,93,94,95,96,97,98], have also been produced. Actually, these mutants pioneered the application of site-directed mutagenesis to the study of PSI [88,89,90]. The main target of these studies was also the identification of the ligands and the interaction of the $F_{X}$-binding domain with the PSI acceptor side subunits. Most of these mutants resulted, however, in a loss or very low level of PSI accumulation, in centres lacking or having a heavily modified $F_{X}$ centre [88,89,90], as well as in altered binding of PsaC and the neighbouring subunits [89,90,91,92,93,94,95,96,97,98]. This, in turn, restricted the possibility of studying the effect of the specific mutations on forward electron transfer, so the characterisation of these modified reaction centres was then relatively limited [94,95,96,97,98]. Although mutants leading to perturbations of the $F_{X}$ properties are certainly interesting, they would lead to the simultaneous alteration of factors governing three ET events: the reduction of this cluster by both phylloquinones and its oxidation by $F_{A}$. Here, then, the discussion will be initially focused on changes in the redox potential of $F_{A}$ ($E_{F_{A}}^{0}$), since the $F_{X}\to F_{A}$ reaction follows immediately after the phylloquinones in the ET cascade but is not expected to modify the uphill reaction rates directly. Yet, any alteration in the $F_{A}$ redox potential leads to changes in both $\Delta G_{F_{X}\to F_{A}}^{0}$ and $\Delta G_{F_{A}\to F_{B}}^{0}$, albeit in the opposite direction; as the driving force for one reaction increases, the other decreases.

**Figure 4:**
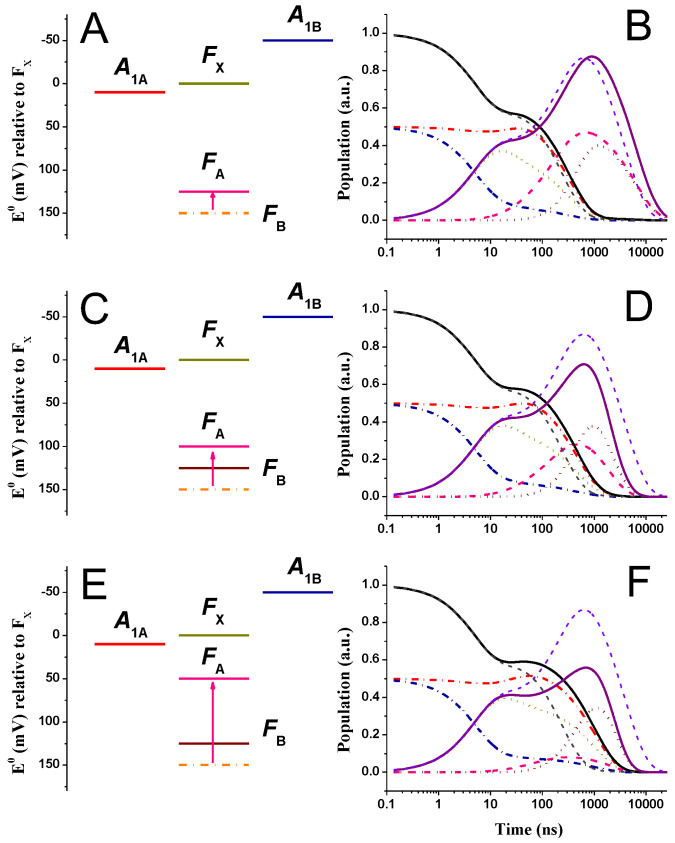
(**A**,**C**,**E**) Energetic schemes showing the $E_{F_{A}}^{0}$ perturbations (altered value, pink line) with respect to the reference model being the “weak driving force” scenario for $A_{1}^{-}$ oxidation. The reference $E_{F_{A}}^{0}$ potential is indicated by orange dash-dotted lines. (**B**,**D**,**F**) Simulated population evolution of the individual redox-active cofactors, resulting from $E_{F_{A}}^{0}$ perturbations. $A_{1A}^{-}(t)$: red dash-dotted line; $A_{1B}^{-}(t)$: blue dash-dotted line; $F_{X}^{-}(t)$: golden dotted line; $F_{A}^{-}(t)$: pink dashed line; $F_{B}^{-}(t)$: burgundy dotted line. The population evolutions of $A_{1,tot}^{-}(t)$ (black line) and $FeS_{tot}^{-}(t)$ (purple line) are also shown and compared with the simulation for the reference scenario (grey and violet dashed lines for $A_{1,tot}^{-}(t)$ and $FeS_{tot}^{-}(t)$, respectively).

**Figure 5:**
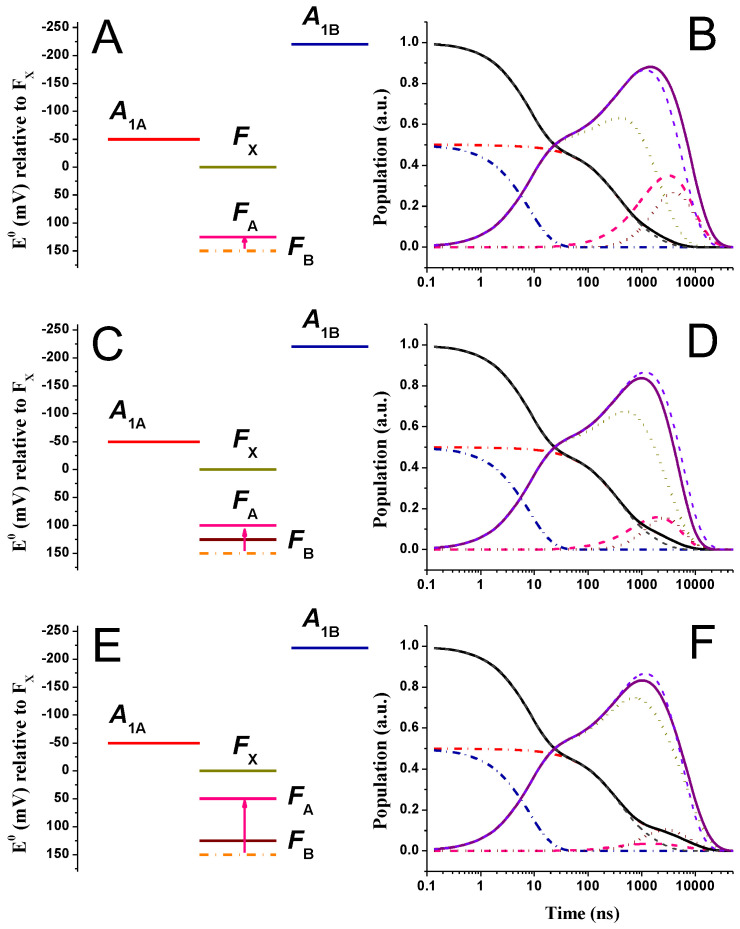
(**A**,**C**,**E**) Energetic schemes showing the $E_{F_{A}}^{0}$ perturbations (altered value, pink line) with respect to the reference model being the “large driving force” scenario for $A_{1}^{-}$ oxidation. The reference $E_{F_{A}}^{0}$ potential is indicated by orange dash-dotted lines. (**B**,**D**,**F**) Simulated population evolution of the individual redox-active cofactors, resulting from $E_{F_{A}}^{0}$ perturbations. $A_{1A}^{-}(t)$: red dash-dotted line; $A_{1B}^{-}(t)$: blue dash-dotted line; $F_{X}^{-}(t)$: golden dotted line; $F_{A}^{-}(t)$: pink dashed line; $F_{B}^{-}(t)$: burgundy dotted line. The population evolutions of $A_{1,tot}^{-}(t)$ (black line) and $FeS_{tot}^{-}(t)$ (purple line) are also shown and compared with the simulation for the reference scenario (grey and violet dashed lines for $A_{1,tot}^{-}(t)$ and $FeS_{tot}^{-}(t)$, respectively).

Figure 4 shows the simulated kinetics for all redox cofactors described in the energetic/kinetic model, in which $E_{F_{A}}^{0}$ was shifted by −25 mV (Figure 4A,B), −50 mV (Figure 4C,D), and −100 mV (Figure 4E,F), within the “weak driving force” scenario for phyllosemiquinone oxidation. For the −25 mV shift, $F_{A}$ and $F_{B}$ are isoenergetic; for larger potential shifts, the driving force for $F_{B}$ reduction (by $F_{A}$) increases progressively, whereas that of $F_{A}$ reduction by $F_{X}^{-}$ decreases accordingly but always remains favourable from a thermodynamic point of view. Figure 5 shows simulations for the same alterations of $E_{F_{A}}^{0}$, but within the “large driving force” phyllosemiquinone oxidation scheme. The simulations of Figure 4 and Figure 5 show some significant differences in the ET kinetics when equal perturbations are applied to the driving forces of reactions, which are otherwise described by basically the same parameters (Figure 2 and Figure 3).

In the “weak driving force” scheme, together with the modification of the population evolutions of $F_{X,A,B}^{-}$, which are expected since the rate constants associated with these reactions are directly affected by the $F_{A}$ redox potential perturbations, significant alterations of the $A_{1,tot}^{-}$ oxidation kinetics are also simulated ($\tau_{av,A_{1}^{-}}$ was 258, 360, and 840 ns vs. ~180 ns in the reference energetic scenario). This is particularly clear for the simulated $A_{1A}^{-}$ kinetics: due to the increase in the value of the lifetime that dominates its oxidation, it is simulated as 340 and 530 ns and 1 μs for the $E_{F_{A}}^{0}$ perturbations discussed above vs. 284 ns in the initial scenario. The kinetics of $A_{1,tot}^{-}$ oxidation became progressively slower as the rate of $F_{X}^{-}\to F_{A}$ became slower (7.2 × 10^−3^, 5.9 × 10^−3^, and 2.1 × 10^−3^ ns^−1^ vs. 1 × 10^−2^ ns^−1^ in the starting scenario) because of the decrease in driving force, $\Delta G_{F_{X}\to F_{A}}^{0}$. However, regardless of the exact shift of the $F_{A}$ redox potential, $F_{X}^{-}$ reduction remained concerted with that of $A_{1,tot}^{-}$ and, on average, slowed down by the same extent ($\tau_{av,F_{X}^{-}}^{d}$ = 326 ns, 413 ns, and 1 μs vs. 199 ns in the reference system). On the other hand, $F_{A}^{-}$ oxidation became progressively faster (as the potential of the cofactor became more reductive), and so did the oxidation of $F_{B}^{-}$, as conditions shifted from endergonic (Figure 2) to exergonic (Figure 4). The maximal population of these cofactors decreased (${\left[F_{\left(X+A+B\right)}^{-}\right]}^{\max}$ = 0.86, 0.70, and 0.53 vs. 0.86 in the reference) with an increase in free energy for the last inter-protein ET step. The effect is clearly more pronounced for the maximal $F_{A}^{-}$ reduction level (${\left[F_{A}^{-}\right]}^{\max}$ = 0.45, 0.26, and 0.06 vs. 0.64 in the reference system).

In contrast, in the “large driving force” scheme, the modifications of the $F_{A}^{-}$ oxidation potential, together with the resulting changes in driving forces associated with the reactions involving this cofactor, appear to affect almost exclusively inter-Fe-S electron transfer reactions, whereas the kinetics of $A_{1,tot}^{-}$ oxidation remain almost unaffected (Figure 5). The only significant effect on $A_{1}^{-}$ oxidation concerned the amplitude of a minor component displaying a long lifetime (~1.5 μs), which was also simulated for the reference system (Figure 3). Its value increased significantly upon $E_{F_{A}}^{0}$ perturbation (τ = 3.7, 5.2, and 14 μs, respectively, vs. 1.4 μs in the reference system). The large value of this lifetime, even when having a small associated amplitude (<10% of total), has a marked impact on the estimation of $\tau_{av,A_{1}^{-}}$ (which was 680 ns, 875 ns, and 2.2 μs in the $F_{A}$-perturbed vs. ~370 ns in the reference energetic system). It should be noted that, to the best of our knowledge, this slow phase has never been detected experimentally. It is clearly connected to the explicit consideration of $F_{A}^{-}\to F_{B}$ reaction, as it is not simulated otherwise ([48,63,70] and Section S1, Table S1). Kinetic coupling in this scenario becomes significant, as the rate of $F_{X}^{-}\to F_{A}$ transfer (8.8 × 10^−4^ ns^−1^) is almost the same as that of $F_{A}^{-}\to F_{B}$ (7.7 × 10^−4^ ns^−1^) and, especially, of the reverse $F_{B}^{-}\to F_{A}$ reaction (1.5 × 10^−4^ ns^−1^). This is not the case for the “weak driving force” scenario due to the lower reorganisation energy (0.7 vs. 1 eV) required to describe the $A_{1}^{-}\to F_{X}$ and $F_{X}^{-}\to F_{A}$ reactions. Thus, when the μs-phase(s) is omitted from the calculations of $\tau_{av,A_{1}^{-}}$ in the “large driving force” simulations, the values of this parameter were almost unaffected by changes of $E_{F_{A}}^{0}$, varying from 122 ns in the reference system to 139 ns for the largest potential shift of −100 mV considered in the simulations of Figure 5.

By comparing Figure 4 and Figure 5, it is clear that the effect of rendering $F_{A}$ potential more reductive is far more pronounced on the ET kinetics involving all iron–sulphur clusters in the case of the “large driving force” scenario for $A_{1}^{-}$ oxidation. The cofactor showing the largest change in ET kinetic is $F_{X}^{-}$, whose oxidation/reduction dynamics became progressively slower, with average decay lifetimes of 3.8, 5.2, and 13 μs compared to 2.7 μs in the reference system. When decreasing the driving force for $F_{X}^{-}$ oxidation, its depopulation became progressively overlapped with that of $F_{A,B}^{-}$, and its maximal population increased from 0.59 in the reference system to 0.69, 0.72, and 0.79 as $F_{A}$ became more reductive. The maximal cumulative population of the Fe-S clusters did not change significantly, indicating that the increase in transient $F_{X}^{-}$ population level was accompanied by a parallel decrease in that of $F_{A+B}^{-}$ (Figure 5).

**Figure 6:**
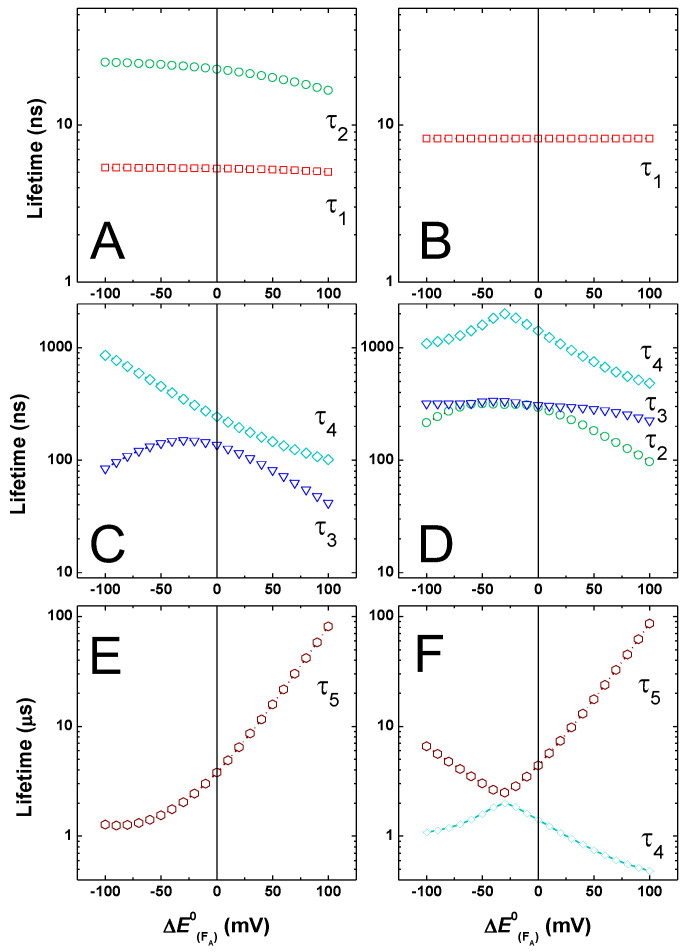
Simulated lifetimes resulting from the variation of $E_{F_{A}}^{0}$ (±100 meV), starting from the “weak” (**A**,**C**,**E**) and “large” (**B**,**D**,**F**) driving force models for $A_{1}^{-}$ oxidation. (**A**,**B**) τ < 50 ns; (**C**,**D**) 50 ns < τ < 1 μs; (**E**,**F**) τ > 1 μs. $\tau_{1}$: red squares; $\tau_{2}$: green circles; $\tau_{3}$: blue triangles; $\tau_{4}$: cyan diamonds; $\tau_{5}$: burgundy hexagon. In panel (**F**), $\tau_{4}$ (cyan diamonds) is re-plotted to facilitate comparison with $\tau_{5}$.

To obtain a more comprehensive picture of the effects resulting from perturbations of the $F_{A}$ redox potential, and in turn the $\Delta G_{F_{X}\to F_{A}}^{0}$ and $\Delta G_{F_{A}\to F_{B}}^{0}$ free energies, the value of $E_{F_{A}}^{0}$ was changed in ±100 mV intervals with respect to the reference systems. The effect of tuning the $E_{F_{A}}^{0}$ redox potential on the simulated lifetimes is presented in Figure 6, starting from the initial reference being either the “weak” (Figure 6A,C,E) or “large” driving force (Figure 6B,D,F) energetics for $A_{1}^{-}$ oxidation. In Figure 6, lifetimes values were separated into classes for ease of presentation and comparison, so that Figure 6A,B show τ < ~50 ns, Figure 6C,D show ~50 ns < τ < ~1 μs, and Figure 6E,F show τ > ~1 μs. Since the lifetimes change upon $F_{A}$ perturbation, this classification is not strict but refers to the dominant behaviour of a given lifetime component.

The values of τ < 50 ns are not greatly affected by any perturbation of $E_{F_{A}}^{0}$ in the ±100 mV interval considered. As regards these lifetimes, $\tau_{1}$ and $\tau_{2}$ in the reference “weak driving force” and $\tau_{1}$ only in the reference “large driving force” energetic scheme reflect principally $A_{1B}^{-}$ oxidation, which is exergonic in both. The simulated kinetics of Figure 4 and Figure 5 show the prediction of little alterations in this kinetic phase when modifying successive ET reactions.

For lifetime values in the 50 ns < τ < ~1 μs range (Figure 6C,D), starting-model-dependent variations are predicted instead. In the starting “weak driving force” case, $A_{1A}^{-}$ oxidation is dominated by the value of $\tau_{4}$, whose simulated values increase with the increase of $\Delta G_{F_{X}\to F_{A}}^{0}$, i.e., with the lowering of the reaction driving force (Figure 6C). The $\tau_{3}$ lifetime in this energetic configuration relates principally to ET reactions amongst the Fe-S clusters, and its reference value of ~160 ns is in agreement with experimental estimation [30,33,34]. Its dependence on the variation of $F_{A}$ redox potential is not monotonic, consistent with it being determined by the kinetic coupling of reactions influenced by both $\Delta G_{F_{X}\to F_{A}}^{0}$ and $\Delta G_{F_{A}\to F_{B}}^{0}$.

In the starting “large driving force” energetic scheme, $A_{1A}^{-}$ oxidation is dominated by the value of $\tau_{3}$ instead, which is largely independent of the redox potential of $F_{A}$. As discussed for the simulations of Figure 5, the two main phases of $A_{1}^{-}$ oxidation remain largely unaffected by perturbing $E_{F_{A}}^{0}$, even in the large ±100 mV range, within this reference scenario (Figure 6D). This is consistent with the phylloquinone oxidation beeing (mainly) kinetically decoupled from the successive ET steps. For this initial energetic configuration, $\tau_{2}$ displays a dependence on $E_{F_{A}}^{0}$ variation similar to the one simulated for $\tau_{3}$ in the “weak driving force” model, albeit its starting value is slightly larger (~250 ns, due to the slightly larger value of $\lambda_{tot}$, i.e., 1 eV vs. 0.9 eV). This component shall then also be taken as principally reflecting transfer between Fe-S clusters. Since it is not simulated when $F_{A}$ and $F_{B}$ are not explicitly considered ([48] and Section S1), it most likely reflects ET transfer between terminal acceptors. This simulated lifetime is in general agreement with the inter 4Fe-4S clusters electron transfer estimated by the Nuclear Magnetic Resonance methods in the ferredoxin of *Chromatium vinosum* (~300 ns), which is structurally analogous to the PsaC subunit [99]. The value of $\tau_{4}$ is also larger in the reference “large driving force” scheme with respect to the “weak driving force” configuration. Its initial value of ~1 μs is the same as the one simulated when $F_{A}$ and $F_{B}$ are not explicitly taken into account ([48] and Section S1) and, therefore, reflects mainly $F_{X}^{-}$ oxidation. This lifetime shows a non-monotonic dependence on $E_{F_{A}}^{0}$, with an apparent discontinuity point at the −40 mV potential shift (Figure 6D), which is for a decrease in $F_{X}^{-}$ oxidation driving force (less exergonic) and an increase in the driving force (more exergonic) for $F_{A}^{-}$ oxidation by $F_{B}$.

Different behaviours with respect to shifts in the $E_{F_{A}}^{0}$ value are also simulated for the slowest lifetime ($\tau_{5}$). In the reference “weak driving force” $A_{1}^{-}$ oxidation, $\tau_{5}$ shows a quasi-monotonic dependence, becoming faster as the driving force for $F_{A}^{-}$ oxidation by $F_{B}$ increases. For the largest favourable energetic configuration, the value of this lifetime approaches the inverse of the output from the system $k_{out}^{-1}$ ~ 1 μs (Figure 6E). In the “large driving force” $A_{1}^{-}$ oxidation framework, $\tau_{5}$ has a more complex dependence, with the lifetime also becoming faster for increased $F_{A}^{-}\to F_{B}$ oxidation driving forces. However, similarly to $\tau_{4}$, which is also re-plotted in Figure 6F for direct comparison, a trend discontinuity point at the ~−40 mV potential shift is simulated. Under these energetic conditions, the decrease in driving force for $F_{X}^{-}$ oxidation dominates over the increased driving force for the successive ET steps.

In both cases, the slowest values simulated, especially for conditions in which $F_{A}^{-}\to F_{B}$ is significantly endergonic, indicate that the equilibration between the terminal electron acceptors can significantly slow down ET to the diffusible acceptors carries (e.g., ferredoxin). A slow $F_{X}^{-}\to F_{A}$ ET reaction would have similar consequences, as shown by the $\tau_{5}$ dependence of Figure 6F as well as the simulations of Figure 4. The latter effect, to our knowledge, has not previously been explicitly considered, even though it might have important implications for photosystem functionality.

**Figure 7:**
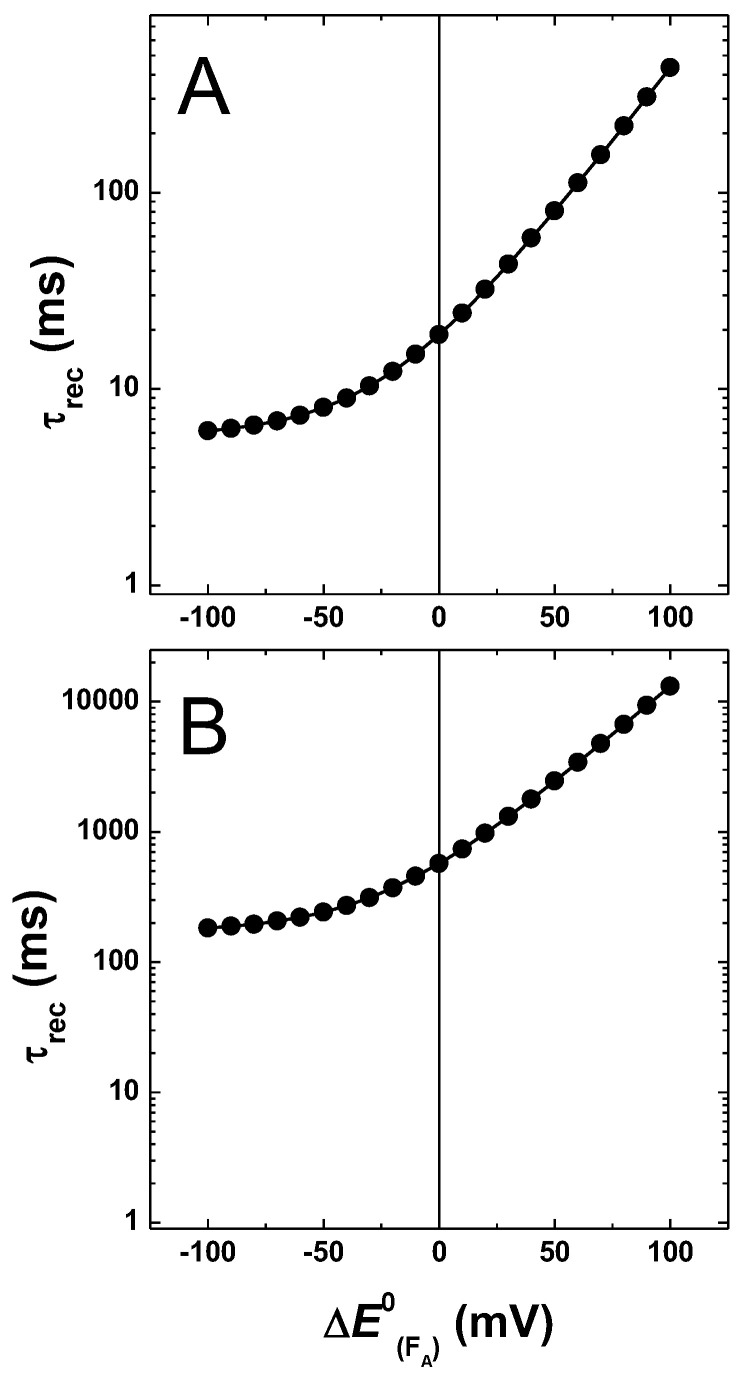
Simulated recombination lifetimes resulting from variation of $E_{F_{A}}^{0}$ (±100 meV), starting from the “weak” (**A**) and “large” (**B**) driving force models for $A_{1}^{-}$ oxidation.

Figure 7 shows the dependence on the simulated recombination reactions, i.e., when $k_{out}$ = 0. In both energetic scenarios, the lifetime for charge recombination displays a positive correlation with the increase in driving force for the $F_{X}^{-}\to F_{A}$ reaction. For values of $E_{F_{A}}^{0}$ that yields $\Delta G_{F_{A}\to F_{B}}^{0}$ < 0 (i.e., ~<−25 mV shift), the recombination lifetime depends only weakly on the exact value of $\Delta G_{F_{X}\to F_{A}}^{0}$.

However, as the $F_{A}^{-}\to F_{B}$ transfer enters the endergonic regime ($\Delta G_{F_{A}\to F_{B}}^{0}$ >> 0), the recombination lifetimes display almost an exponential response with respect to the decrease of $\Delta G_{F_{X}\to F_{A}}^{0}$, i.e., an increase in driving force for the forward and a decrease in backward reactions, in agreement with the back-population of $F_{X}^{-}$ imposing important kinetic limitations on these recombination reactions.

### 2.4. Effect of Changing the Driving Force of $F_{A}^{-}$ Oxidation ($\Delta G_{F_{A}\to F_{B}}^{0}$)

The driving force for $F_{A}^{-}$ oxidation can be modulated, without directly affecting the energetics of $F_{X}^{-}$, by modifying the redox potential of $F_{B}$ ($E_{F_{B}}^{0}$).

**Figure 8:**
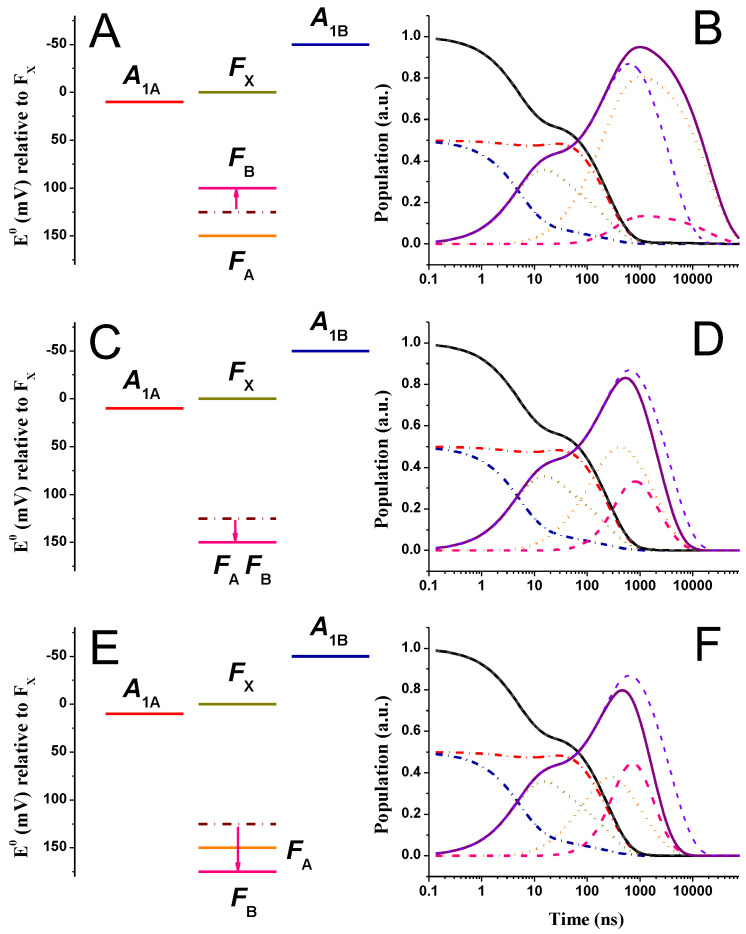
(**A**,**C**,**E**) Energetic schemes showing the $E_{F_{B}}^{0}$ perturbations (altered value, pink line) with respect to the reference model being the “weak driving force” scenario for $A_{1}^{-}$ oxidation. The reference $E_{F_{B}}^{0}$ potential is indicated by burgundy dash-dotted lines. (**B**,**D**,**F**) Simulated population evolution of the individual redox-active cofactors, resulting from $E_{F_{B}}^{0}$ perturbations. $A_{1A}^{-}(t)$: red dash-dotted line; $A_{1B}^{-}(t)$: blue dash-dotted line; $F_{X}^{-}(t)$: golden dotted line; $F_{A}^{-}(t)$: orange dotted line; $F_{B}^{-}(t)$: pink dashed line. The population evolutions of $A_{1,tot}^{-}(t)$ (black line) and $FeS_{tot}^{-}(t)$ (purple line) are also shown and compared with the simulation for the reference scenario (grey and violet dashed lines for $A_{1,tot}^{-}(t)$ and $FeS_{tot}^{-}(t)$, respectively).

**Figure 9:**
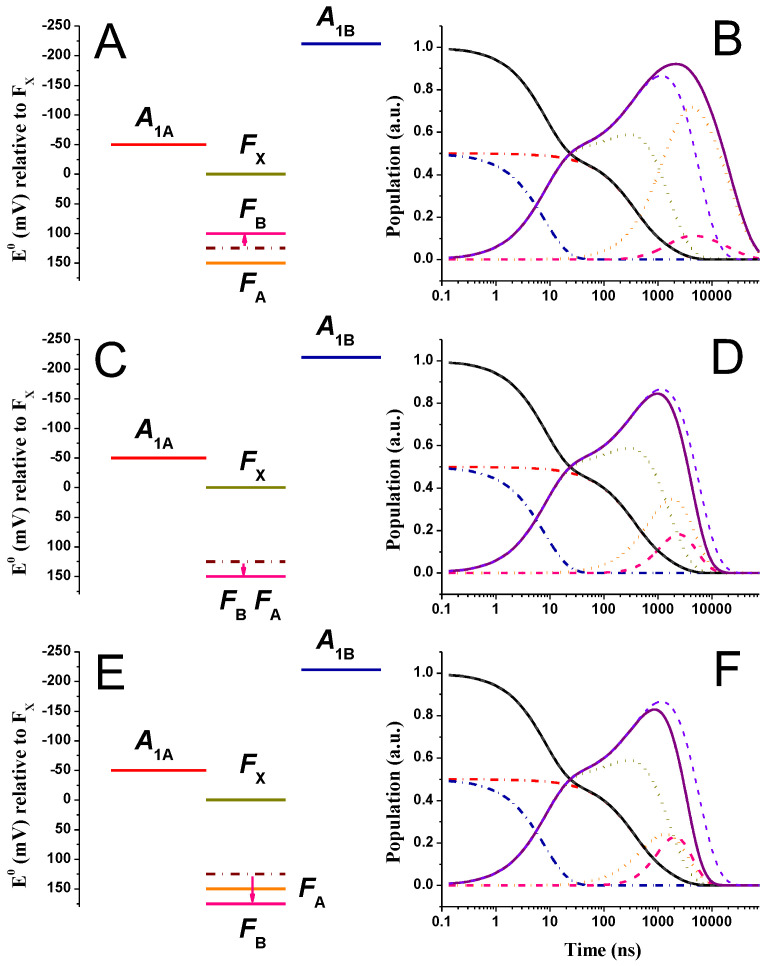
(**A**,**C**,**E**) Energetic schemes showing the $E_{F_{B}}^{0}$ perturbations (altered value, pink line) with respect to the reference model being the “large driving force” scenario for $A_{1}^{-}$ oxidation. The reference $E_{F_{B}}^{0}$ potential is indicated by burgundy dash-dotted lines. (**B**,**D**,**F**) Simulated population evolution of the individual redox-active cofactors, resulting from $E_{F_{B}}^{0}$ perturbations. $A_{1A}^{-}(t)$: red dash-dotted line; $A_{1B}^{-}(t)$: blue dash-dotted line; $F_{X}^{-}(t)$: golden dotted line; $F_{A}^{-}(t)$: orange dotted line; $F_{B}^{-}(t)$: pink dashed line. The population evolutions of $A_{1,tot}^{-}(t)$ (black line) and $FeS_{tot}^{-}(t)$ (purple line) are also shown and compared with the simulation for the reference scenario (grey and violet dashed lines for $A_{1,tot}^{-}(t)$ and $FeS_{tot}^{-}(t)$, respectively).

**Figure 10:**
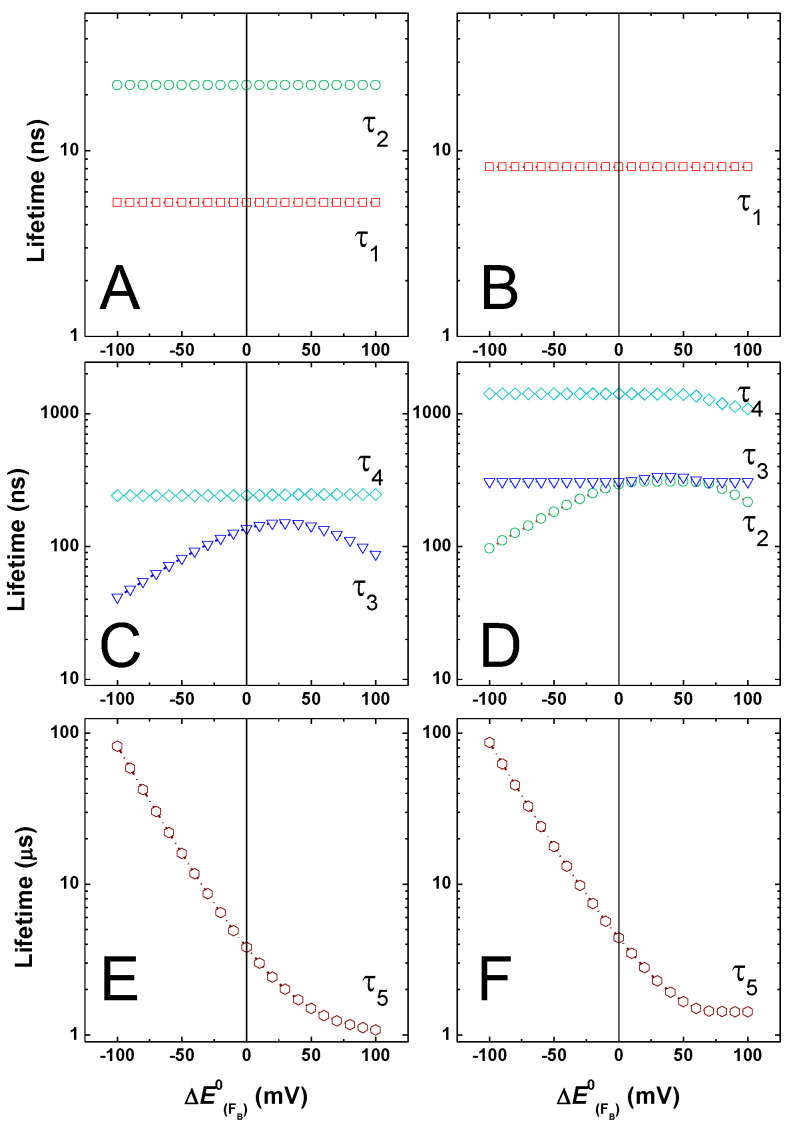
Simulated lifetimes resulting from the variation of $E_{F_{B}}^{0}$ (±100 meV), starting from the “weak” (**A**,**C**,**E**) and “large” (**B**,**D**,**F**) driving force models for $A_{1}^{-}$ oxidation. (**A**,**B**) τ < 50 ns; (**C**,**D**) 50 ns < τ < 1 μs; (**E**,**F**) τ > 1 μs. $\tau_{1}$: red squares; $\tau_{2}$: green circles; $\tau_{3}$: blue triangles; $\tau_{4}$: cyan diamonds; $\tau_{5}$: burgundy hexagon.

Figure 8 (“weak driving force” energetics) and Figure 9 (“large driving force” energetics) show the simulated kinetics of all redox cofactors considered in the models, upon shifting the $F_{B}$ potential by −25 mV (Figure 8B and Figure 9B), +25 mV (Figure 8D and Figure 9D), and +50 mV (Figure 8F and Figure 9F). In this case, for the −25 mV shift, $F_{A}^{-}\to F_{B}$ becomes more endergonic ($\Delta G_{F_{A}\to F_{B}}^{0}$ = +50 meV); for the +25 mV $E_{F_{B}}^{0}$ shift, $F_{A}$ and $F_{B}$ are isoenergetic ($\Delta G_{F_{A}\to F_{B}}^{0}$ = 0 meV); and for the +50 mV shift, $F_{A}^{-}$ oxidation is exergonic ($\Delta G_{F_{A}\to F_{B}}^{0}$ = −25 meV). The impact of exploring a ±100 mV variation of $E_{F_{B}}^{0}$ on the lifetimes, describing ET in PSI, is shown in Figure 10.

Comparison of the simulations presented in Figure 8 and Figure 9 indicates that irrespective of the reference energetic model used to describe $A_{1}^{-}$ oxidation, alterations affecting selectively $\Delta G_{F_{A}\to F_{B}}^{0}$ do not affect the phyllosemiquinone oxidation kinetics, as they are, in both cases, basically indistinguishable from the respective reference system. The same holds true for wider changes in $E_{F_{B}}^{0}$ of ±100 mV, since the values of $\tau_{1}$, $\tau_{2}$, and $\tau_{4}$ (Figure 10A,C), which dominate $A_{1}^{-}$ oxidation in the “weak” driving force scenario, are barely affected by changes in $\Delta G_{F_{A}\to F_{B}}^{0}$. The same is observed for $\tau_{1}$ and $\tau_{3}$ that dominates $A_{1}^{-}$ oxidation in the “large driving force” $A_{1}^{-}$ oxidation framework (Figure 10B,D). The value of the $\tau_{4}$, which is also associated with a small-amplitude-μs $A_{1}^{-}$ oxidation phase in this energetic configuration, barely depends on $\Delta G_{F_{A}\to F_{B}}^{0}$. On the other hand, as expected, changing $\Delta G_{F_{A}\to F_{B}}^{0}$ affects the kinetics of electron transfer between the Fe-S clusters, especially the temporal evolutions of $F_{A}^{-}$ and $F_{B}^{-}$, whereas that of $F_{X}^{-}$ is only slightly perturbed. In brief, the overall reduced population of $F_{A}$ increases with the decrease in driving force for its oxidation by $F_{B}$, and vice versa, so that with the decrease of $\Delta G_{F_{A}\to F_{B}}^{0}$, the temporal evolution of the reduced state of this cofactors becomes less concerted and, basically, sequential for large driving forces.

Interestingly, the values of $\tau_{3}$ (“weak driving force” reference energetics, Figure 10C) and $\tau_{2}$ (“large driving force” reference energetics, Figure 10D) show a non-monotonous dependence on variation of $\Delta G_{F_{A}\to F_{B}}^{0}$, with the slowest values simulated upon $F_{A}$ and $F_{B}$ becoming close to iso-energetic in both cases. These behaviours are similar to those simulated for variations in the $E_{F_{A}}^{0}$, which also affect $\Delta G_{F_{A}\to F_{B}}^{0}$.

The lifetime showing the greatest dependence on $E_{F_{B}}^{0}$ is the one representing the effective exit from the system ($\tau_{5}$). An almost monotonous decrease (decay deceleration) is predicted upon the progressively decreasing $\Delta G_{F_{A}\to F_{B}}^{0}$, for values of $E_{F_{B}}^{0}$ under which, the $F_{A}^{-}\to F_{B}$ reaction falls in the endergonic regime. A softer dependence is simulated instead for the large exergonic regimes, i.e., for $F_{B}$ potential shifts >~60–80 mV (Figure 10E,F). Under the latter conditions, the value of $\tau_{5}$ (at least in the “weak driving force” scenario) approaches that of $k_{out}^{-1}$ (~1 μs).

It is, however, worth mentioning that the simulated lifetimes, especially for large positive shifts of the $F_{B}$ potential, should be taken as indicative only, since the exit from the system ($k_{out}$), which provides a phenomenological description of ferredoxin/flavodoxin reduction, is held at a constant value in the simulations. This process is, at least in the case of ferredoxin, also an inter-FeS cluster electron transfer reaction that depends on the free energy difference between $F_{B}$ and the 2Fe-2S cluster bound to ferredoxin, whose redox potential was determined to be ~−410 mV (e.g., refs. [64,100,101]). The estimated driving force in both reference scenarios is, therefore, rather large, with $\Delta G_{F_{B}\to Fd}^{0}$ ~ −150 meV. However, as the $F_{B}$ potential increases, the absolute value of $\Delta G_{F_{B}\to Fd}^{0}$ becomes smaller. The free energy contraction is expected to slow ferredoxin reduction and, at the same time, render it substantially reversible. Collectively, this shall cause an overall decrease in the effective exit constant, $k_{out}$. These aspects should be taken into account to obtain more accurate simulations of terminal PSI electron transfer cofactor kinetics when interacting with physiological carriers.

**Figure 11:**
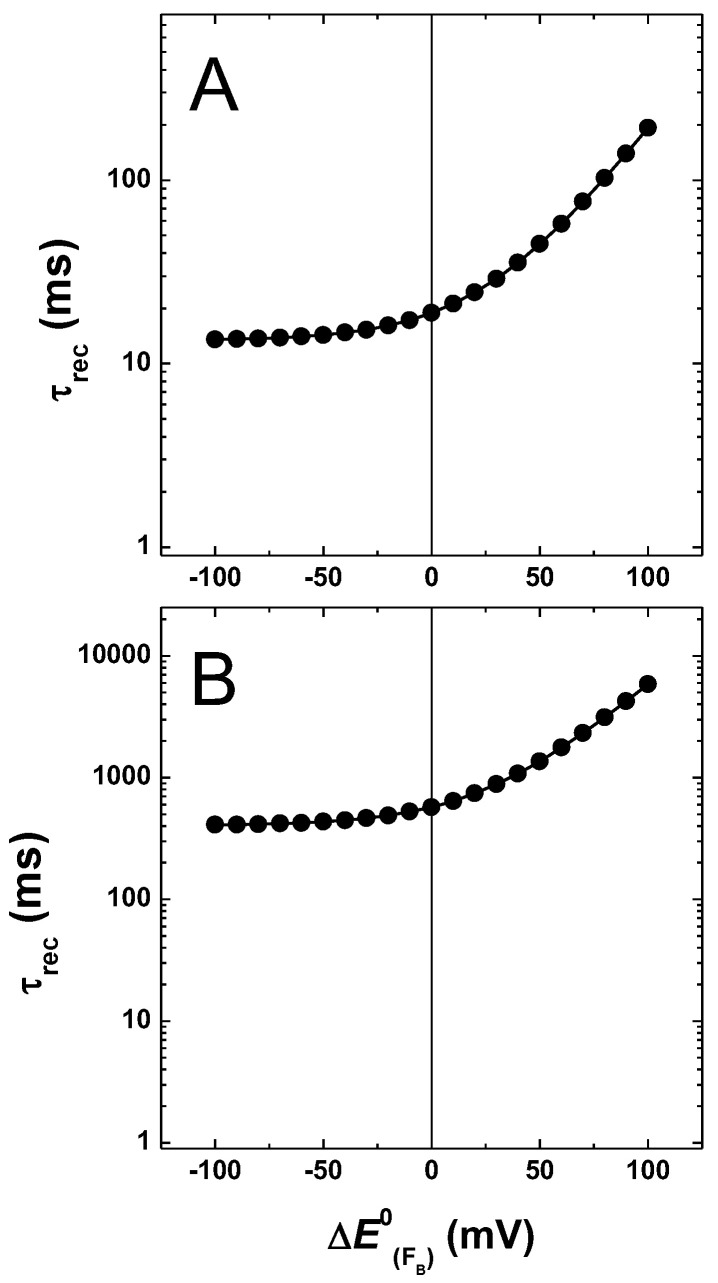
Simulated recombination lifetimes resulting from the variation of $E_{F_{B}}^{0}$ (±100 meV), starting from the “weak” (**A**) and “large” (**B**) driving force models for $A_{1}^{-}$ oxidation.

Figure 11 shows the simulated recombination in the absence of an exit from the system. Regardless of the energetics associated with $A_{1}^{-}$ oxidation, the kinetics of charge recombination displayed a weak dependence on $\Delta G_{F_{A}\to F_{B}}^{0}$, maintaining the value simulated for the reference scenario, with shifts in $E_{F_{B}}^{0}$ of less than about ~60 mV. A significant increase in the simulated recombination lifetimes is, however, predicted when $F_{A}^{-}\to F_{B}$ enters the large endergonic regime, $\Delta G_{F_{A}\to F_{B}}^{0}$ > 60 mV. The overall response of charge recombination kinetics is similar to but less pronounced than the one predicted upon perturbations of $E_{F_{A}}^{0}$, which affects both $\Delta G_{F_{A}\to F_{B}}^{0}$ and $\Delta G_{F_{X}\to F_{A}}^{0}$ (Figure 7), albeit in contrasting fashion. This indicates that, although the repopulation of $F_{X}^{-}$ imposes the largest kinetic limitations to charge recombination, back-population of $F_{A}^{-}$ will also slow this reaction when its oxidation by $F_{B}$ becomes largely favourable.

## 3. Materials and Methods

### 3.1. Electron Transfer Dynamics Simulations

Kinetic simulations were performed as described previously [12,48,49]. In brief, the population evolutions of the redox cofactors considered in the kinetic model (as schematically shown in Figure 1 and Figure S9 where are all rates are given explicitly) were obtained by solving a system of ordinary differential equations, which, in compact matrix form, is defined by $\dot{P}(t)=K_{i}\cdot P(t)$. P(t) is a column vector containing as its elements the cofactors population evolutions: $\left(A_{1A}^{-}(t),A_{1B}^{-}(t),\ldots ,F_{B}^{-}(t)\right)$, whereas $\dot{P}(t)$ is a column vector containing their first derivatives with respect to time. $K_{i}$ is a square matrix, frequently referred to as the rate matrix, which has as its elements the rate constants between pairs of electron donors and acceptors (see Supplementary Materials Section S5 for the extended form of the system of differential equations and of the rate matrix $K_{i}$). The index *i* corresponds to the number of cofactors (states) considered in the modelling. The system of linear differential equation has a general solution of the form $P(t)=\sum_{J=1}^{i}c_{j}V_{j}e^{\zeta_{j}t}$, where $\zeta_{j}$ and $V_{j}$ are the eigenvalues and the eigenvectors, respectively, which diagonalise the matrix $K_{i}$; and $c_{j}$ are a set of scalars that need to be determined according to some given initial conditions, which in this case were $A_{1A}^{-}(0)$ = $A_{1B}^{-}(0)$ = 0.5 and $F_{X}(0)$ = $F_{A}(0)$ = $F_{B}(0)$ = 0. The general solution of the system corresponds to the empirical linear combination of weighted exponentials, which is frequently employed to describe the experimental kinetics. Thus, the eigenvalues, which are univocally determined by the eigen-decomposition of $K_{i}$, reflect in the experimentally observed lifetimes ($\tau_{j,obs}$) through the relation $\tau_{j,obs}=-\zeta_{j}^{-1}$. The product $c_{j}V_{j}$ relates only indirectly to the experimental pre-exponential amplitudes. The modelled amplitudes represent “weighted” molar fractions and describe only the changes in the relative cofactor concentration over time, whereas the experimental values are also determined by the monitored properties of the cofactors (e.g., the wavelength-dependent extinction coefficient, fluorescence yield/spectra, and so on).

### 3.2. Electron Transfer Rates Description

The link with theory is established by using an explicit physical description of the rate constants rather than setting their values as free simulation parameters. To this end, here we adopted the description for a pair of donor-acceptor molecules, originally derived by Hopfield [36,102], when considering a single (mean) nuclear mode coupled to the ET event:

(1) $$\begin{array}{l} k_{D\to A}=\frac{2\pi }{\hbar }{\left|H_{DA}\right|}^{2}\frac{1}{\sqrt{2\pi \sigma^{2}}}e^{-\frac{{\left(\Delta G_{DA}^{0}+\lambda_{tot}\right)}^{2}}{2\sigma^{2}}}\\ \sigma^{2}=\lambda_{tot}\hbar \bar{\omega }\coth\frac{\hbar \bar{\omega }}{2k_{B}T} \end{array}$$

where $\lambda_{tot}$ is the total reorganisation energy, $\bar{\omega }$ is the angular frequency of the mean coupled nuclear mode, ℏ is the Dirac constant, $k_{B}$ is the Boltzmann constant, *T* is the temperature, and ${\left|H_{DA}\right|}^{2}$ is the electronic coupling term. ${\left|H_{DA}\right|}^{2}$ can be to a good level approximated as ${\left|H_{DA}\right|}^{2}={\left|H_{0}\right|}^{2}e^{-\beta (X_{DA}-3.6)}$, where ${\left|H_{0}\right|}^{2}$ is the maximal value of the electronic coupling at wavefunctions overlap, β is a damping term associated with the probability of tunnelling the potential barrier, *X_DA_* is the edge-to-edge cofactor distance (in Å), and 3.6 represents a correction for the van der Waals radii. When $\hbar \bar{\omega }$ << $k_{B}T$, $\sigma^{2}=2\lambda k_{B}T$, and the expression therefore simplifies, yielding the semi-classical relation derived by Marcus [35].

The total reorganisation energy $\lambda_{tot}$ results from two components referred to as the inner-($\lambda_{i}$) and outer-sphere ($\lambda_{o}$) reorganisations, with the first term being dominated by the displacements of the nuclei of the cofactors and the second term by the reconfiguration of the surrounding medium. The outer shell reorganisation, for redox centres embedded in a homogenous medium, can be described as follows [35,36]:

(2) $$\lambda_{o}=\frac{e^{2}}{4\pi \varepsilon_{0}}(\frac{1}{\varepsilon_{opt}}-\frac{1}{\varepsilon_{s}})(\frac{1}{2r_{A}}+\frac{1}{2r_{D}}-\frac{1}{R_{\mathrm{DA}}})$$

where e is the elementary charge of the electron, $\varepsilon_{0}$ is the electric permittivity of vacuum, $\varepsilon_{opt}$ and $\varepsilon_{s}$ are the so-called optical (frequency dependent, in the high-frequency limit) and static relative permittivity of the medium, $r_{A}$ and $r_{D}$ are the radii of the acceptor and donor molecules, and $R_{\mathrm{DA}}$ is the centre-to-centre distance between them. Hence, the value of $\lambda_{o}$ is minimal for small differences between $\varepsilon_{opt}$ and $\varepsilon_{s}$, which is considered to be the case for most protein environments [66,67]. On the other hand, $\lambda_{o}$ increases upon increasing the donor–acceptor separation and tends to a close to distance-independent value when $R_{\mathrm{AD}}$ ≫ $r_{A}$,$r_{D}$. The cofactor separation dependency is however less dominant than the one due to differences in the relative permittivity characteristics of the medium.

The determination of the reorganisation energy is often challenging from an experimental perspective, especially in biological systems. The analysis is further complicated when several electron transfer reactions take place in a redox chain. The experimental estimation of $\lambda_{tot}$ is usually extracted from temperature-dependence studies of systems in which the redox properties and/or free energy differences are known in advance or can be determined electrochemically. Hence, the number of experimentally determined values of $\lambda_{tot}$ is not extremely extended, whereas a wider range of synthetic models or mimicking systems has been investigated instead (e.g., refs. [67,103] and references therein). Different computational approaches for the prediction of the reorganisation energy values, relying on the availability of structural data, have been also developed (for reviews see e.g., refs. [104,105]), but tend to be computationally expensive. Simpler approaches, such as those introduced by Dutton, Moser, and coworkers (e.g., refs. [37,65,106,107]), based on the use of more probable/frequent values in redox proteins, provide sensible and straightforward alternatives for $\lambda_{tot}$ estimation. These kinds of consensus values represent reasonable approximations only, serving as a rational initial guess for the system description. Hence, together with the other simplifications that are often employed in kinetic modelling, including the present study, the simulated predictions should be considered to represent a semi-qualitative/quantitative rather than exact description of cofactors’ oxidation-reduction kinetics.

Considering the accuracy limits just discussed, the simulations were performed adopting values of ${\left|H_{0}\right|}^{2}$ ~ 1.3 × 10^−3^ eV^2^ (e.g., refs. [36,48,49]) and β = 1.38 Å^−1^ [37] common to all reactions. The edge-to-edge distances $X_{\mathrm{DA}}$ are obtained from the high-resolution crystallographic model of PSI at 2.5 Å resolution [3], and these are the only relevant parameters extracted from the structure. The reaction-specific values of $\Delta G_{DA}^{0}$, $\hbar \bar{\omega }$, and $\lambda_{tot}$ adopted to describe a given electron transfer reaction are discussed in the Results section. In general, a parsimony criterion that allows to keep the number of variable parameters as low as possible is adopted.

### 3.3. Average and Mean Lifetimes

It is often convenient to compare the average ($\tau_{av}$) and mean ($\tau_{m}$) lifetime obtained from the simulations, rather than comparing the individual pre-exponential amplitudes ($p_{i}$) and lifetimes. These parameters are defined, for each of the levels (cofactors) considered, as follows:

(3) $$\tau_{av}=\frac{\sum_{i}p_{i}\tau_{i}}{\sum_{i}p_{i}}$$

Since the sum at the denominator is zero for all cofactors that have no initial population, two other derived parameters associated with it are considered for these cofactors, the average rise ($\tau_{av}^{r}$) and depopulation ($\tau_{av}^{d}$) lifetimes. These are obtained by performing the summations in Equation (3), considering only the pre-exponential terms with negative ($p_{i-}$) or positive ($p_{i+}$) amplitudes, respectively.

The mean lifetimes represent the first moment of the population evolution, which is described by a linear combination of exponentials and is defined for each cofactor as follows:

(4) $$\tau_{m}=\frac{\sum_{i}p_{i}\tau_{i}^{2}}{\sum_{i}p_{i}\tau_{i}}$$

which is always defined regardless of the initial amplitudes of the cofactors.

## 4. Conclusions

In this work, the effect of altering the redox potential of the terminal acceptors of PSI, the iron–sulphur clusters, $F_{A}$ and $F_{B}$, has been explored employing theory-based kinetic model simulations. This approach allows us to predict the electron transfer kinetics between the Fe-S clusters, which has proven cumbersome to address experimentally by spectroscopic methods due to the characteristics of these cofactors and the spectral crowding occurring in large chromophore-pigment super-complexes, such as PSI. The effect of altering $F_{A}$ and $F_{B}$ redox reactivity was comparatively explored by considering two energetic scenarios for ET reactions involving the upstream redox cofactors, the iron–sulphur cluster $F_{X}$ and the phylloquinones $A_{1A}$ and $A_{1B}$, as the latter two are better characterised by time-resolved spectroscopic approaches. From the presented analysis, it is possible to infer the following:

(i). Tuning the $F_{A}$ redox potential, together with an alteration of the ET kinetics directly involving this redox centre, also affects the phyllosemiquinone oxidation kinetics, particularly the slow kinetic phase of this process that is dominated by the depopulation of $A_{1A}^{-}$. Significant kinetic perturbations are predicted within the “weak driving force” scenario for $A_{1}^{-}$ oxidation by $F_{X}$. Tuning $E_{F_{A}}^{0}$ has instead a limited effect on $A_{1}^{-}$ oxidation kinetics when considering the “large driving force” energetic scheme for these cofactors’ oxidation reactions. Since both energetic schemes describe adequately several measured parameters, they can both be considered realistic. A more in-depth analysis of mutants affecting $F_{A}$ coordination in the PsaC subunit, some of which have already been reported (e.g., refs. [78,79,80,81,82,83,84]) but not studied with sufficient (ns) temporal resolution, is expected to shed further light on the energy gap between $A_{1(A/B)}$ and $F_{X}$, especially since the redox potential of the modified $F_{A}$ cofactor shall be accessible by direct electrochemistry and henceforth correlated to the kinetic perturbation. Similar approaches have already been explored and proven feasible (e.g., refs. [78,79,80]). However, as already discussed, the effect of the mutations on the kinetics of forward ET reactions has not been investigated in detail yet.
(ii). Tuning the $F_{B}$ redox potential is predicted to affect almost exclusively ET involving the iron–sulphur clusters instead, especially the oxidation kinetics of $F_{A}^{-}$ and $F_{B}^{-}$, without significantly impacting on the $A_{1}^{-}$ oxidation kinetics, regardless of the energetic scenario employed to describe the latter reaction.
(iii). Alterations of the $F_{B}$ redox potential, which have also been reported for mutants affecting its coordination site in the PsaC subunit (e.g., refs. [76,77,78,79,80,81,82,83,84,85,86,87]), can, however, help in elucidating the actual energetics of ET between the terminal iron–sulphur clusters, which might be inferred from differences in both the recombination rates and forward ET to ferredoxin, in comparison with a similar perturbation of the $F_{A}$ redox potential.
(iv). The ~180 ns kinetic phase experimentally retrieved in investigations of $A_{1}^{-}$ oxidation kinetics [30,33,34,62,63] appears to be associated principally with $F_{A}^{-}$ oxidation by $F_{B}$, whereas it was previously assigned to $F_{X}^{-}$ oxidation by $F_{A}$ [30]. The ~180 ns lifetime appears to be a significant contribution also in the $F_{X}^{-}$ oxidation kinetics, indicating that $F_{A}^{-}$ oxidation by $F_{B}$ is not fully kinetically decoupled from upstream reactions. This lifetime is correctly predicted in the “weak driving force” $A_{1}^{-}$ oxidation scenario, whereas a slight overestimation (~300 ns) is obtained in the “large driving force” energetic scheme.
(v). The need to consider $\lambda_{tot}$ ~ 1 eV in the “large driving force” $A_{1}^{-}$ oxidation scenario led to simulating a relatively slow $F_{X}^{-}$ oxidation, which couples to a small-amplitude $A_{1A}^{-}$ oxidation phase in the μs windows. This phase has not been detected experimentally either because it is not present or not resolvable. Multiple pieces of evidence suggest that $F_{X}^{-}$ oxidation shall proceed in the sub-μs scale. This can be taken as an indication that $\lambda_{tot}$ ~ 1 eV represents an upper limit/overestimation of this parameter. The calculated recombination rates, which are limited by the back-population of $F_{X}^{-}$, argue in the same direction, as they are overestimated, in general, by about an order of magnitude within the “large driving force” scenario (see Sections S2 and S3 of the Supplementary Materials for further discussion and simulations).
(vi). Uphill ET from $F_{A}^{-}$ to $F_{B}$ can significantly slow down donation from the terminal electron acceptor(s) to diffusible carriers, at least when pre-bound complexes are formed, i.e., the speed of electron transfer to ferredoxin (or other acceptors) might not depend solely on the rate of transfer from $F_{B}^{-}$ to the redox-active centre in the acceptor molecules, particularly if the intrinsic rate constant for this reaction is as fast as, or comparable with, the electron transfer kinetics involving the terminal electron acceptors within the photosystem.

## Figures and Tables

**Table 1:** Rate-defining parameters and simulated ET kinetic for the “weak driving force” energetic scenario.

Rate Determining Parameters						Electron Transfer Simulated Parameters							
Reaction	$X_{DA}$ (Å)	$\Delta G_{DA}^{0}$ (eV)	$\lambda_{t,DA}$ (eV)	${\bar{\omega }}_{DA}$ (eV/ cm^−1^)	$k_{D\to A}$ (ns^−1^)	τ (ns)	Amplitudes						
							$p_{A_{1A}}$	$p_{A_{1B}}$	$p_{F_{X}}$	$p_{F_{A}}$	$p_{F_{B}}$	$p_{A_{1\mathrm{tot}}}$	$p_{FeS_{\mathrm{tot}}}$
$A_{1A}^{-}\to F_{X}$	9.1	0.01	0.700	0.022 (175)	0.0181	5.26	0.0754	0.382	−0.489	0.033	0.000	0.457	−0.457
$A_{1B}^{-}\to F_{X}$	9.0	−0.05	0.700	0.046 (375)	0.145	22.5	−0.173	0.051	0.172	−0.051	0.003	−0.123	0.123
$F_{X}^{-}\to F_{A}$	11.6	−0.15	0.700	0.034 (275)	0.0126	136	0.0035	0.000	0.001	−0.295	0.336	0.004	0.042
$F_{A}^{-}\to F_{B}$	9.5	0.025	0.900	0.034 (275)	0.0019	243	0.587	0.067	0.312	−0.489	−0.628	0.654	−0.806
$F_{B}^{-}\to$	–	–	–	–	0.00100	3808	0.0068	0.001	0.005	0.803	0.290	0.008	1.098

Summary of the parameters used for the simulation of forward electron transfer in reference PSI within the “weak driving force” scenario for $A_{1}^{-}$ oxidation. The rate-defining parameters that are common to all forward electron reactions are the electronic coupling matrix element ${\left|H_{0}\right|}^{2}$ = 1.3 10^−3^ eV^2^, the barrier camping factor β = 1.34 Å^−1^, and the temperature *T* = 290 K, which are defined in Equation (1). The table also lists the lifetimes ($\tau_{i}$) and associated amplitudes ($p_{i}$) describing the population evolution of each of the cofactors described in the model. The total amplitude associated with the electron transfer kinetics of phylloquinones ($p_{A_{1\mathrm{tot}}}$) and iron–sulphur centres ($p_{FeS_{\mathrm{tot}}}$) are also presented in the table. The initial conditions for the amplitude simulations were $A_{1A}^{-}(0)=A_{1B}^{-}(0)=0.5$ and zero on all other cofactors considered.

**Table 2:** Rate-defining parameters and simulated ET kinetic for the “large driving force” energetic scenario.

Rate Determining Parameters						Electron Transfer Simulated Parameters							
Reaction	$X_{DA}$ (Å)	$\Delta G_{DA}^{0}$ (eV)	$\lambda_{t,DA}$ (eV)	${\bar{\omega }}_{DA}$ (eV/ cm^−1^)	$k_{D\to A}$ (ns^−1^)	τ (ns)	Amplitudes						
							$p_{A_{1A}}$	$p_{A_{1B}}$	$p_{F_{X}}$	$p_{F_{A}}$	$p_{F_{B}}$	$p_{A_{1\mathrm{tot}}}$	$p_{FeS_{\mathrm{tot}}}$
$A_{1A}^{-}\to F_{X}$	9.1	−0.05	1.0	0.022 (175)	0.00270	8.15	0.002	0.500	−0.504	0.0035	0.0000	0.501	−0.501
$A_{1B}^{-}\to F_{X}$	9.0	−0.22	1.0	0.046 (375)	0.12249	294.7	0.0020	0.000	−0.003	0.3891	−0.550	0.002	−0.164
$F_{X}^{-}\to F_{A}$	11.6	−0.15	1.0	0.034 (275)	0.00085	307.3	0.307	0.000	−0.414	−0.343	0.650	0.307	−0.107
$F_{A}^{-}\to F_{B}$	9.5	0.025	1.0	0.034 (275)	0.00077	1411	0.187	0.001	0.912	−1.292	−0.468	0.188	−0.847
$F_{B}^{-}\to$	–	–	–	–	0.00100	4415	0.002	0.000	0.001	1.242	0.367	0.002	1.619

Summary of the parameters used for the simulations of forward electron transfer in reference PSI within the “large driving force” scenario for $A_{1}^{-}$ oxidation. The rate-defining parameters that are common to all forward electron reactions are the electronic coupling matrix element ${\left|H_{0}\right|}^{2}$ = 1.3 10^−3^ eV^2^, the barrier camping factor β = 1.34 Å^−1^, and the temperature *T* = 290 K, which are defined in Equation (1). The tables also list the lifetimes ($\tau_{i}$) and associated amplitudes ($p_{i}$) describing the population evolution of each of the cofactors described in the model. The total amplitude associated with the electron transfer kinetics of phylloquinones ($p_{A_{1\mathrm{tot}}}$) and iron–sulphur centres ($p_{FeS_{\mathrm{tot}}}$) are also presented in the table. The initial conditions for the amplitude simulations were $A_{1A}^{-}(0)=A_{1B}^{-}(0)=0.5$ and zero on all other cofactors considered.

## Data Availability

The dataset is available upon request from the authors.

## Supplementary Materials

The supporting information can be downloaded at: https://www.mdpi.com/article/10.3390/ijms25189795/s1.
