# Supplementary material for: Thermodynamic Factors Controlling Electron Transfer among the Terminal Electron Acceptors of Photosystem I: Insights from Kinetic Modelling
Source: Int J Mol Sci. 2024 Sep 10;25(18):9795. doi: 10.3390/ijms25189795 (PMC11432928; doi:10.3390/ijms25189795)
Supplement: Supplementary file 1 [file ijms-25-09795-s001.zip › ijms-3171608-supplementary.pdf]

Thermodynamic factors controlling electron transfer amongst the terminal electron acceptors of Photosystem I: insight from kinetic modelling.

Stefano Santabarbara[1*] & Anna Paola Casazza[1]

[1]Photosynthesis Research Unit, Consiglio Nazionale delle Ricerche, Via A. Corti 12, 20133 Milano, Italy

Supplementary Information

Section S1. Comparison of model simulations for three- and five-cofactors kinetic model, and influence of recombination reactions to P_{700}^{+}

Figure S1 shows the simulations of electron transfer kinetics resulting from considering explicitly three redox cofactors only, A_{1A}, A_{1B} and F_X, and within either the weak driving force (A, B) or large driving force (C, D) scenarios. Since the F_A and F_B cofactors, do not appear in these calculations, the rate of F_X^- oxidation represents the effective output from the systems.

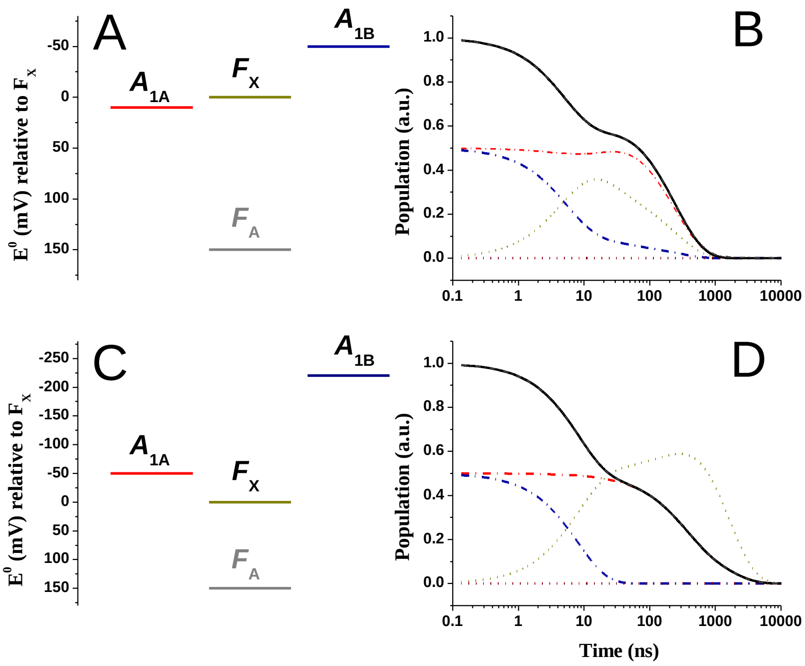

Figure S1. Simulations of forward electron transfer kinetics within the "weak diving force" (A, B) and "large driving force" (C, D) schemes when considering only the phylloquinones $A_{1A/B}$ and F_X. The population evolution of $A_{1A}^-(t)$ (dashed-dot red line), $A_{1B}^-(t)$ (dashed-dot blue line) and $F_X^-(t)$ (dotted golden line) are shown in panels B and D, together with the total population evolutions of $A_{1,tot}^-(t) = A_{1A}^-(t) + A_{1B}^-(t)$ (black line). The simulations of $A_{1,tot}^-(t)$ obtained from the extended five-

cofactor schemes are also shown as dash-dotted grey line. These are almost indistinguishable from those resulting from the restricted three-cofactor ones.

The kinetic simulations were performed employing all the relevant parameters associated with the individual rate constants already discussed in the main body of the text. In this case, together with the difference in energetics shown in panels A and C, the only relevant difference is that λ_{tot} was 0.7 for the weak driving force and 1 eV for the large driving force scenario. With respect to previous reports in which an analogous three-cofactor system has been presented and discussed (ref **[44]** of the main text, same thereafter), the recombination between each cofactor and P_{700}^{+} is here included. However this does not influence the simulation of the forward electron transport kinetics since the recombination rates are some order of magnitude slower that the forward ones. Furthermore, the simulated kinetics of $A_{1,tot}^{-}$ are not significantly affected by explicitly considering the F_{A} and F_{B}, as the simulated kinetics are fundamentally indistinguishable when considering the terminal iron-sulphur clusters implicitly or explicitly.

The main parameters resulting from the simulations are listed in Table S1.

Table S1. Simulation outcomes resulting from the three- and five-cofactor kinetic scheme.

**Weak Driving Force**						**Large Driving Force**					
Three-states			Five-states			Three-states			Five-states		
τ (ns)	$p_{A_{1,tot}}$	p_{F_X}	τ (ns)	$p_{A_{1,tot}}$	p_{F_X}	τ (ns)	$p_{A_{1,tot}}$	p_{F_X}	τ (ns)	$p_{A_{1,tot}}$	p_{F_X}
5.26	0.46	-0.49	5.26	0.46	-0.49	8.15	0.50	-0.50	8.15	0.50	-0.50
22.5	-0.12	0.17	22.5	-0.12	0.17	307	0.31	-0.42	295	0.00	0.00
244	0.67	0.32	136	0.00	0.00	1420.	0.19	0.92	307	0.31	-0.41
			243	0.65	0.31				1411	0.19	0.91
			3808	0.01	0.00				4415	0.00	0.01

Comparison of the outcomes from simulations considering either three- or five-cofactor kinetic/energetic schemes, starting either for the weak-driving force or the large-driving force scenario. Shown are the resulting lifetimes together with the relative amplitudes (pre-exponential factors) coupled to each lifetime for the population evolution of $A_{1,tot}^{-}$ ($p_{A_{1,tot}}$) and F_{X}^{-} (p_{F_X}).

The comparison of lifetimes reported in Table S1 allows to straightforwardly identify the component directly related to reactions involving the iron-sulphur clusters F_{A} and F_{B}, since, irrespectively on the reference energetic scheme considered, three out of the five

simulated lifetimes are fundamentally the same. The F_A and F_B electron transfer specific lifetimes are the 136 ns and the 3.8 μs, in the weak driving force model, and the 296 ns and 4.4 μs in the large driving force scenario. It is also worth noting that, in both scenarios, these lifetimes have negligibly small amplitudes coupled to either $A_{1,tot}^-$ or F_X^- oxidation, again confirming their assignment to transfer between F_A and F_B (the 136 and 295 ns, the exact value depending on the energetic scheme) and oxidation of both cluster by external acceptors, for the longest-lived component.

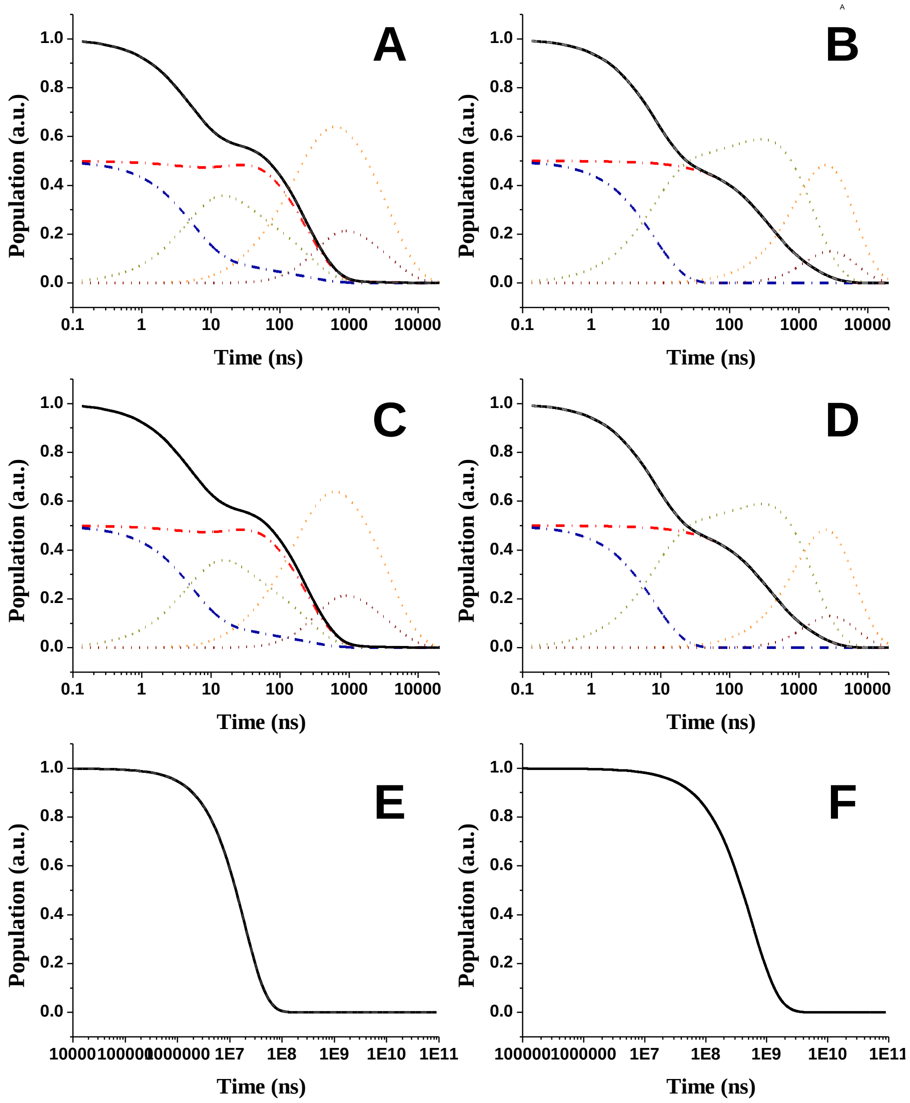

Figure S2. Simulations of electron transfer kinetics within the "weak diving force" (**A, C**) and the "large driving force" (**B, D**) schemes when omitting either all the recombination rate to P_{700}^+ (**A, B**), or including only the recombination between P_{700}^+ and $A_{1A/B}^-$ (**C, D**). Population evolutions: $A_{1A}^-(t)$ (dashed-dot red line), $A_{1B}^-(t)$ (dashed-dot blue line), $F_X^-(t)$ (dashed golden line), $F_A^-(t)$ (dashed orange line) and), $F_B^-(t)$ (dashed burgundy line), and $A_{1,tot}^-(t) = A_{1A}^-(t) + A_{1B}^-(t)$ (black line). Panels **E**

and **F** shows the recombination kinetics in the absence of an exit from F_{B}^{-}, corresponding to the kinetic schemes of panels **C** and **D**, respectively. The simulations of $A_{1,tot}^{-}(t)$ and of the recombination reactions obtained from the extended five-cofactor schemes, including the recombination reactions, are also shown as dash-dotted grey line and are almost indistinguishable from simulations is which they are neglected.

In **Figures S2** are shown the population evolutions of forward electron transfer kinetics, utilising the same kinetic scheme as described in the main text, but omitting all recombination reactions to P_{700}^{+} from the calculations. Both for weak driving force (**Figure S2A**) or the large driving force (**Figure S2B**) the inclusion of the P_{700}^{+} recombination reactions has fundamentally no effect on the forward electron transfer. Further **Figures S2C** and **S2D** shows the simulated forward reactions when omitting all recombination reactions from the FeS clusters, but maintaining the recombination between P_{700}^{+} and $A_{1A/B}^{-}$. Although this has, again, no influence on the forward reactions, it allows to estimate the recombination under conditions in which the exit from the system is suppressed. **Figures S2E** and **S2F** show that the simulated recombination kinetics are fundamentally unaffected when the direct recombination between P_{700}^{+} and all the FeS is neglected, indicating that under these conditions (absence of both diffusing acceptors and donors) recombination takes place from A_1^{-} and that the recombination kinetics are limited by the uphill repopulation of this cofactor from F_X.

Section S2. Comparison of model simulations for coupling with nuclear modes of different frequencies

To further explore the possible effect of some of the specific values employed in the kinetic models used as references for the "weak"" and "large" driving force scenarios in the simulations of FeS electron transfer, additional simulations were performed also employing different values of the mean nuclear mode coupled to the electron transfer processes ($\bar{\omega}_{DA}$). In the simulations shown in the main text the values, $\hbar\bar{\omega}_{A_{1\mathrm{A}}\to F_{\mathrm{X}}}$ = 21 meV (175 cm^{-1}) and $\hbar\bar{\omega}_{A_{1\mathrm{B}}\to F_{\mathrm{X}}}$ = 47 meV (375 cm^{-1}) were used for the phyllosemiquinone oxidation and $\hbar\bar{\omega}_{DA}$ = 34 meV (275 cm^{-1}), that is the mean of phylloquinone specific modes, was used for all other reactions, *e.g.* electron transfer between FeS clusters and direct recombination to P_{700}^{+}. The values of $\hbar\bar{\omega}_{A_{1\mathrm{A}}\to F_{\mathrm{X}}}$ = 21 meV (175 cm^{-1}) and $\hbar\bar{\omega}_{A_{1\mathrm{B}}\to F_{\mathrm{X}}}$ = 47 meV (375 cm^{-1}) were taken from an experimental investigation of these reactions temperature dependence [**69**] and their

inclusion in the modelling was shown to improve the simulations of the kinetics temperature dependence with respect to considering a dominant low-frequency mode of 20 cm^{-1} **[44]**.

From an extensive survey of electron transfer reactions in proteins, including bacterial reaction centres, it was suggested that a, general, appropriate value for $\hbar\bar{\omega}_{DA}$ would be ~55 meV (450 cm^{-1}) hence larger than the one employed so far in the presented simulations **[37]**. To explore the effect of varying the value of $\hbar\bar{\omega}_{DA}$, **Figure S3** presents the results obtained for the kinetic simulations in which $\hbar\bar{\omega}_{DA}$ = 55 meV was employed for *all* forward and recombination reactions and in both the "weak driving" (**Figure S3A**) and "large driving" (**Figure S3B**) force energetic configurations. In **Figure S3** are also shown the simulation obtained when considering $\hbar\bar{\omega}_{A_{1A}\rightarrow F_X}$ = 21 meV and $\hbar\bar{\omega}_{A_{1B}\rightarrow F_X}$ = 47 meV for A_{1A}^- and A_{1B}^- oxidation and the larger $\hbar\bar{\omega}_{DA}$ = 55 meV for all other reactions.

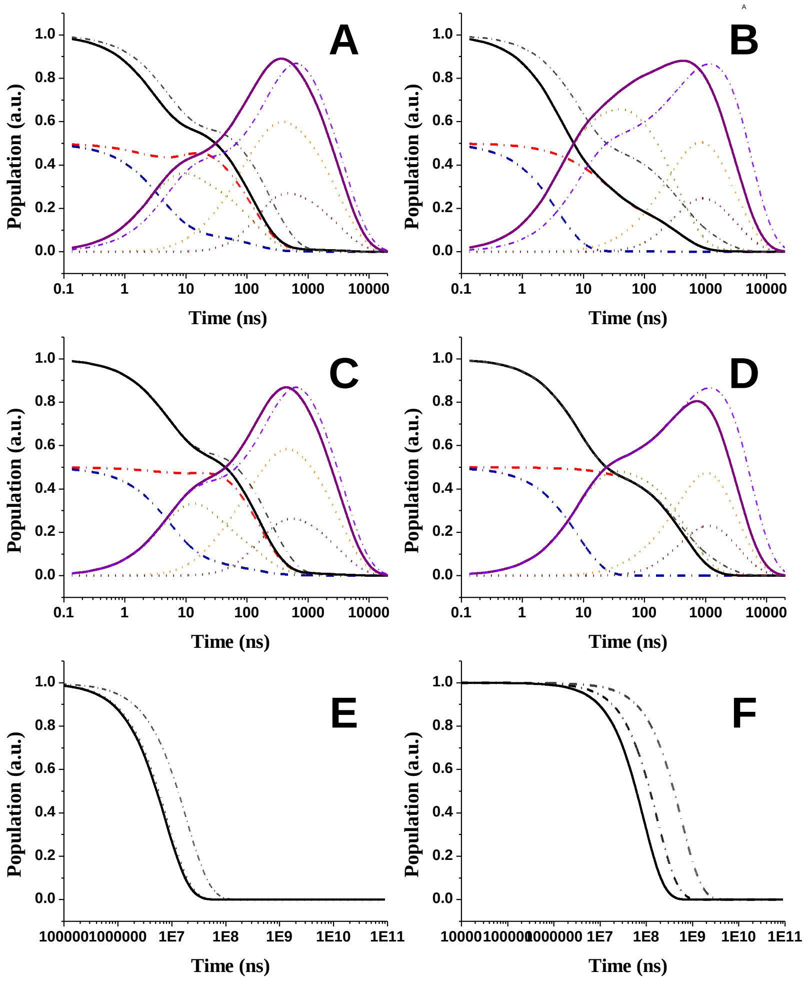

Figure S3. Simulations of forward electron transfer kinetics within the "weak diving force" (**A, C**) and the "large driving force" (**B, D**) schemes when considering $\hbar\bar{\omega}_{DA}$ = 55 meV for all electron transfer reactions (**A, B**) or specific coupling $\hbar\bar{\omega}_{A_{1A}\rightarrow F_X}$ = 21 meV and $\hbar\bar{\omega}_{A_{1B}\rightarrow F_X}$ = 47 meV for A_1^-

oxidation and $\hbar\bar{\omega}_{DA}$ = 55 meV for all the remaining reactions (**C**, **D**). The population evolution: $A_{1A}^{-}(t)$ (dashed-dot red line), $A_{1B}^{-}(t)$ (dashed-dot blue line) and $F_{X}^{-}(t)$ (dot golden line), $F_{A}^{-}(t)$ (dot orange line), $F_{B}^{-}(t)$ (dot burgundy line), $A_{1,tot}^{-}(t) = A_{1A}^{-}(t) + A_{1B}^{-}(t)$ (black line) and $FeS_{tot}^{-}(t) = F_{X}^{-}(t) + F_{A}^{-}(t) + F_{B}^{-}(t)$ (purple line). Also shown are the population evolutions of $A_{1,tot}^{-}(t)$ (dashed-dotted grey lines) and $FeS_{tot}^{-}(t)$ (dash-dotted violet lines) for the reference schemes discussed in the main text. Panels **E** and **F** show the recombination kinetics in the absence of an exit from F_{B}^{-}, corresponding to the kinetic schemes of panels **A** and **B** (black solid lines), and of panels **C** and **D** (black dash-dot lines), respectively. The dash-dotted grey line are the simulations of the recombination reactions in the respective reference systems.

The increase in energy of the nuclear mode causes an acceleration of the electron transfer kinetics of both the phylloquinone oxidation and the electron transfer involving the iron-sulphur cluster as can be seen from the comparison of the population evolutions of $A_{1,tot}^{-}(t)$ and $FeS_{1,tot}^{-}(t)$ in **Figure S3**. This can be related mainly to a direct increase of the electron transfer rates brought about by the larger $\hbar\bar{\omega}_{DA}$ value. In this respect, it is also clear that the overall electron transfer acceleration is more significant when $\hbar\bar{\omega}_{DA}$ is increased for all electron transfer reactions (**Figure S3A** and **B**) than when it is considered for the transfer involving the iron-sulphur clusters (and the recombination reactions) only (**Figures S3C** and **D**).

In the "weak driving force" scenario, when $\hbar\bar{\omega}_{DA}$ is 55 meV for all reactions, the average lifetime of $A_{1,tot}^{-}$ is 126 ns with respect to 188 ns in the reference scheme, mostly due to a decrease in the value of the lifetime that dominates A_{1A}^{-} oxidation from 243 ns in the reference to 123 ns. The kinetics of F_{X}^{-} oxidation are also accelerated, with average lifetimes of 137 ns with respect to 198 ns. When the frequency of the coupled nuclear mode is increased only for forward reactions involving the iron-sulphur clusters, the same trend is observed but is less pronounced. The average lifetime of $A_{1,tot}^{-}$ is simulated as 155 ns while that of F_{X}^{-} becomes 153 ns. In both cases this is also due to a decrease of the 243 ns lifetime in the reference "weak energetic" scheme to 155 ns, which further strengthen the observation that within this energetic pictures, the kinetics of electron transfer of $A_{1,tot}^{-}$, or at least A_{1A}^{-}, and that of F_{X}^{-} are closely linked. Both the lifetime of the slow phase of $A_{1,tot}^{-}$ oxidation as well as τ_{av} are faster than those retrieved experimentally when considering an increase in $\hbar\bar{\omega}_{DA}$, suggesting that a decrease in the driving force of this reaction shall be required to match the experimental values as were for instance obtained when employing the semi-empirical "ruler" relation where this frequency mode is included [7]. Concerning the FeS cluster

reactions, when the $\hbar\bar{\omega}_{DA}$ = 55 meV is considered, it determines a faster rise of the collective population of FeS_{tot}^{-} from 157 ns in the reference scheme, to 98 ns and 72 ns, when $\hbar\bar{\omega}_{DA}$ = 55 meV is specifically associated to inter FeS reactions or all reactions, respectively. This is largely associated to the decrease in value of the 136 ns lifetimes, which is iron-sulphur centres specific, to about 45 ns in both cases. The effect on FeS_{tot}^{-} oxidation is also sizable but somewhat less pronounced, the average lifetimes being 2.8 μs in both case with respect to 3.3 μs in the reference scheme. This also confirms the effect of inter FeS electron transfer in determining also the effective output from the system which is not limited by the exit rate alone.

The acceleration of electron transfer dynamics when coupling a higher frequency mode to all electron transfer steps is even more evident in the "large driving force" scenario (**Figure S3B**), where the average lifetime for $A_{1,tot}^{-}$ oxidation is 100 ns rather than 370 ns in the reference scenario. The most significant effect on the simulated A_{1A}^{-} oxidation is that it becomes markedly biphasic and characterised by lifetimes of 23 and 371 ns, whereas A_{1B}^{-} is dominated by a 3 ns lifetime. When a stronger coupling with nuclear modes is considered for the inter iron-sulphur (and direct recombination reactions) only (**Figure S3D**) the effect is less pronounced, since τ_{av} for $A_{1,tot}^{-}$ oxidation is 233 ns. A_{1A}^{-} oxidation is dominated by a single lifetime of 461 ns and that of A_{1B}^{-} by one of 8.5 ns. Most of the acceleration of $A_{1,tot}^{-}$ oxidation is then due to a decrease in the value of the relative long-lived component (1.4 μs) simulated in the reference scenario, which is substituted by the 461 ns lifetime. This is clearly visible in the tail of $A_{1,tot}^{-}(t)$ population evolutions in **Figure S3D**.

Changes in the dynamics associated to electron transfer amongst the FeS clusters of PSI are also evident upon considering $\hbar\bar{\omega}_{DA}$ = 55 meV. The rise of FeS_{tot}^{-} population becomes significantly faster, with average rise time varying from 835 ns in the reference scenario to 98 ns when increasing the frequency of the mean coupled mode. The larger effect is on the population kinetics of F_{X}^{-} that is simulated as 10 ns rather than 140 ns and its relaxation, which almost coincides with the raise of F_{A+B}^{-}, that becomes 390 ns rather than 1.4 μs in the respective reference scheme. The FeS_{tot}^{-} oxidation also displays a simulated acceleration from 4.8 μs to 4 μs. Similar changes in the kinetics involving the iron-sulphur clusters, although less pronounced, are simulated when $\hbar\bar{\omega}_{DA}$ = 55 meV is considered specifically for these reactions but not for A_{1}^{-} oxidation (**Figure S3D**). The value of F_{A+B}^{-} becomes 228 ns (instead

of 1.4 μs), the acceleration being mainly linked to both the faster oxidation of F_{X}^{-} (560 ns vs 1.4 μs) and the decrease in the lifetimes assigned to the transfer between F_{A} and F_{B} (46 ns vs 1.4 μs). The FeS_{tot}^{-} oxidation displays a simulated acceleration from 4.8 μs to 3 μs, which is even larger than when $\hbar\overline{\omega}_{DA}$ = 55 meV was considered for all reactions.

A significant effect of increasing the value of $\hbar\overline{\omega}_{DA}$ is also simulated for the recombination kinetics in both energetic scenario considered (**Figure S3E** and **F**). In the "weak driving force" configuration the recombination is accelerated to 7.6 ms (general) and 8.2 ms (FeS and recombination only) with respect to 18.9 in the reference scheme and hence is only weakly dependent on considering either a general increase of $\hbar\overline{\omega}_{DA}$ or a specific one coupled to iron-sulphur clusters and direct recombination. In the "large driving force" configuration the effect is however most distinct, with the recombination accelerating to 174 ms when $\hbar\overline{\omega}_{DA}$ = 55 meV is employed for direct recombination and iron-sulphur electron transfer only and to 89 ms when it applied to all reaction with respect to 573 ms in the reference.

The values for the recombination kinetics obtained for the increased value of $\hbar\overline{\omega}_{DA}$ are all relatively close or fall within the relative large spread (10-200 ms) reported experimentally, with the conserved trend that recombination is faster in the weak with respect to the large driving force energetic scheme. For the latter, increasing the value of $\hbar\overline{\omega}_{DA}$ to 55 meV, either to all reactions or to all but A_1^{-} oxidation, leads to a much better agreement with the experimental values for the recombination. On the other hand, the simulations of forward electron transfer kinetics are less consistent with the experimental observation in this case, since whereas the overall increase in A_1^{-} oxidation would still be within the experimental reported values, although at the edge of the faster ones, the ratio of fast:slow phase would change to 0:75:025 (the opposite of what experimentally reported when the slow phase is, if anything, larger) due to contribution of a ~25 ns lifetime which is not present in the reference energetic scheme.

Moreover, for both energetic schemes considered the lifetime that can be associated to electron transfer between F_A and F_B assumes a value of ~ 45 ns when $\hbar\overline{\omega}_{DA}$ = 55 meV. This value appears too small with respect to one observed experimentally of ~140 ns [**30, 33**], even in place of some possible ambiguity on the assignment of this kinetic phase. A better agreement between experimental and simulated kinetics for this lifetime and when considering a mean coupled mode of 450 cm^{-1} would imply a lower driving force of $F_{\mathrm{A}}^{-} \rightarrow F_{\mathrm{B}}$ transfer, *i.e.* a larger positive value of $\Delta G^{0}_{F_A \rightarrow F_B}$.

Section S3. Effect of the value of the total reorganisation energy, λ_{tot}, on the forward electron transfer and recombination kinetics within the "large driving force" A_1^- oxidation model

As discussed in the main text, a satisfactory description of the A_1^- oxidation kinetics is achieved within the "large" driving force model for phylloquinone oxidation, ($\Delta G^0_{A_{1A}\to F_X}$ = –50 meV and $\Delta G^0_{A_{1B}\to F_X}$ = –220 meV (Milanovsky *et al.* **[39]**) when a relatively large value of the total reorganisation energy λ_{tot}=1 eV is considered (for parsimony and homogeneity) for all electron transfer (ET) reactions included in the kinetic model. Within these model parameters the predicted kinetics of F_X^- oxidation (with the minus sign indicating the reduced form of the acceptor, not its net charge) are significantly slower (~ 1 μs) than those assessed experimentally (~50-250 ns). The kinetics of this reaction are well accounted by the "weak" driving force scenario ($\Delta G^0_{A_{1A}\to F_X}$ = +10 meV and $\Delta G^0_{A_{1B}\to F_X}$ = –50 meV), when the same values of free energies for ET between Fe-S clusters, $\Delta G^0_{F_X\to F_A}$ = –150 meV and $\Delta G^0_{F_A\to F_B}$ = +25 meV, and the same mean coupled modes ($\bar{\omega}_{DA}$=34 meV/275 cm^{-1}) utilised in the large driving force scenario are considered. Then, concerning ET amongst Fe-S clusters, the difference between the model simulations reside is the reorganisation energies that for the weak driving were considered to be $\lambda_{t,F_X\to F_A}$=0.7 eV and $\lambda_{t,F_A\to F_B}$ = 0.9 eV. The value of $\lambda_{t,F_X\to F_A}$ was the same as for A_1^- oxidation kinetics, whereas the larger one $\lambda_{t,F_A\to F_B}$ accounts for reaction involving metal centres.

In order to demonstrate the impact of the value of λ_{tot} on the simulation of ET amongst the terminal Fe-S acceptors of PSI within the large driving force scenario, kinetic modelling was performed considering different values of λ_{tot} in the 0.75-0.95 eV interval (the same value common to all reactions), and all other parameters as described in the main text. The simulated ET kinetics are presented in **Figure S4**.

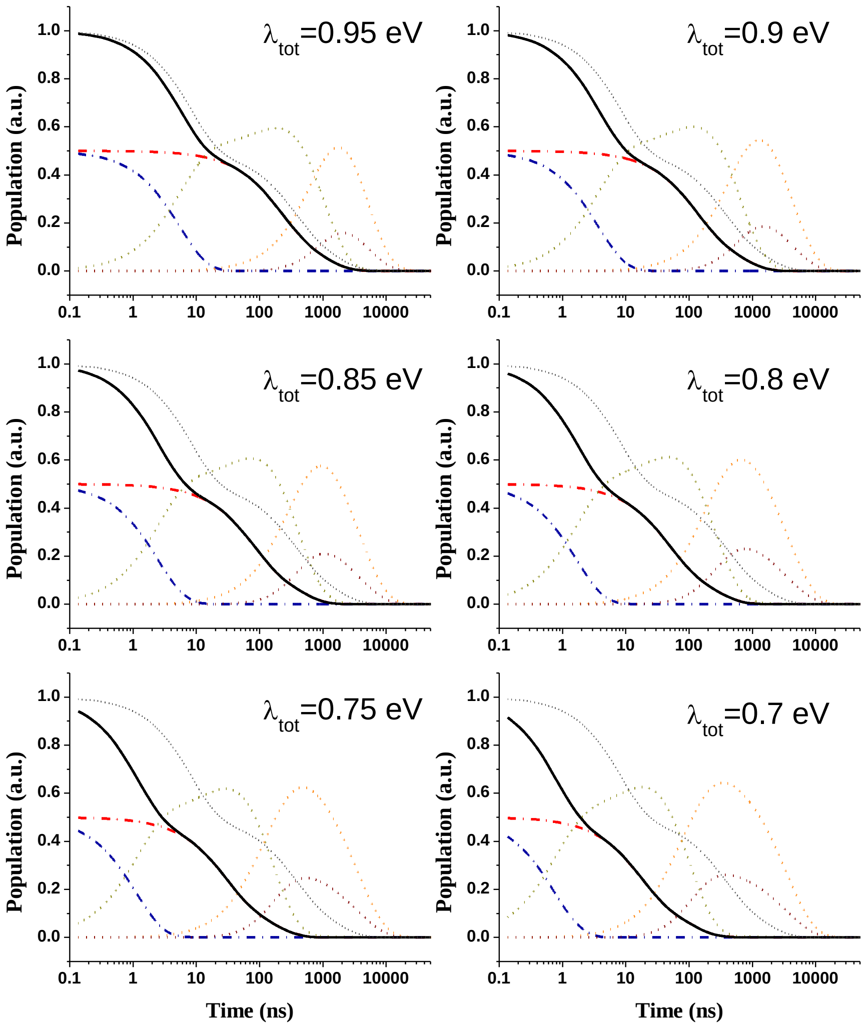

Figure S4. Simulations of forward electron transfer kinetics within the "large driving force scenario" for different values of the total reorganisation energy in the 0.7 – 0.95 eV range. In each simulation the value of λ_{tot} was communal to all reactions considered $A_{1A}^{-}(t)$: dashed-dot red line, $A_{1B}^{-}(t)$ dashed-dot blue line, $F_{X}^{-}(t)$ dotted golden line, $F_{A}^{-}(t)$ dotted orange line, $F_{B}^{-}(t)$ dotted burgundy line. Total population evolutions of $A_{1,tot}^{-}(t) = A_{1A}^{-}(t) + A_{1B}^{-}(t)$ (black line). The dotted grey lines are the simulation of $A_{1,tot}^{-}(t)$ with λ_{tot} = 1 eV as in the main text.

As it may be intuitively expected, decreasing the value of λ_{tot} resulted in an acceleration of *all* forward ET kinetics. Upon considering λ_{tot} <0.85 eV the mean oxidation lifetime of F_{X}^{-} falls within values (~350 ns) that are compatible with the experimental ones. Yet, under these simulation conditions the A_{1}^{-} oxidation is significantly faster than the experimental ones, with the mean oxidation lifetime of A_{1A}^{-} being predicted as 180.5 ns (**Figure S5** shows the dependence of average lifetimes as a function of λ_{tot}) and the dominating lifetime to be 66.5 ns, rather than the 250-300 ns retrieved in experiments. The

longer average lifetime results also from the contribution of a 340 ns lifetime (about 1/3 of the amplitude) which is the dominant phase of F_X^- oxidation.

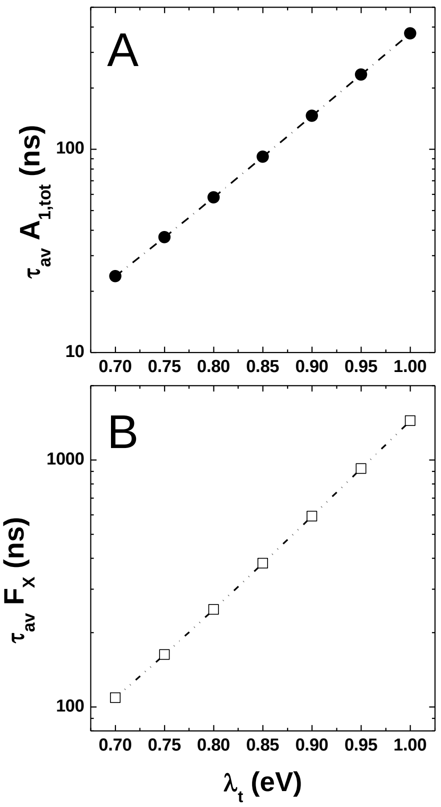

Figure S5. Dependence of the average lifetime of $A_{1,tot}^-$ (A, closed circles) and F_X^- (B, open squares) oxidation on the value of total reorganisation energy, λ_{tot} within the large driving force scenario for A_1^- oxidation. In each simulation, the same value of λ_{tot} was employed for all, forward and recombination, reactions considered.

Further decrease of the value of λ_{tot} leads to predicted A_1^- oxidation which are even further deviates from the experimental values. When considering the same value of λ_{tot} as in the weak driving force scenario (0.7 eV), the oxidation of F_X^- is characterised by an average lifetime of 108 ns, but the average A_1^- oxidation is as fast as 25 ns, almost a factor of ten faster than experimentally determined.

The simulation of charge recombination reactions between P_{700}^+ and the reduced cofactors explicitly considered in the model ($A_{1A/B}^-$ and $F_{X/A/B}^-$) within the large driving force model resulted in predicted lifetimes of ~550 ms, that are approximately from twice to an order of magnitude larger than the experimental values (~10-200 ms). Since, within the kinetic

model discussed here, the limiting step in charge recombination is the energetically uphill repopulation of the F_X^- from $F_{A/B}^-$, a larger value of $\lambda_{t,F_X \to F_A}$ = 1 eV was considered with respect to the "weak" driving force scenario ($\lambda_{t,F_X \to F_A}$ = 0.7 eV) that instead reproduced qualitatively the experimental values. Simulations were performed within the "large driving force" model considering λ_{tot} in the 0.75-1 eV interval (for all reactions) and in the absence of an exit from F_B^- to obtain the charge recombination dynamics. The results are shown in **Figure S6**.

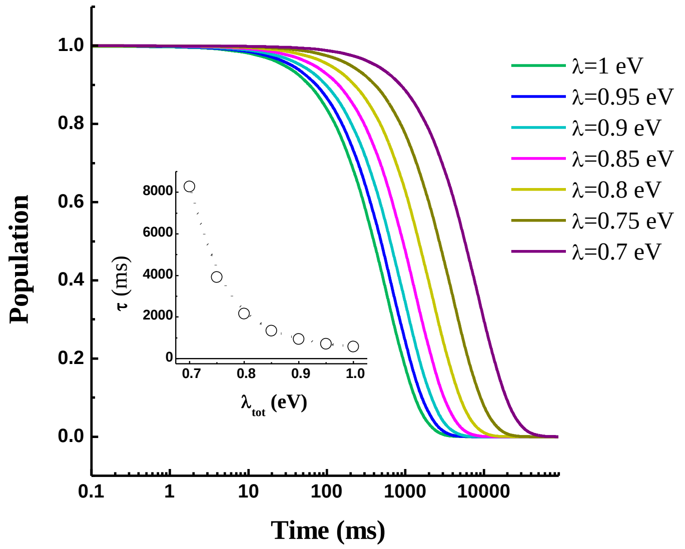

Figure S6. Simulations of recombination kinetics within the "large" driving force model for different values of the total reorganisation energy in the 0.7 – 1 eV range. The inset shows the value of the average recombination lifetime as a function of the reorganisation energy. The same value of λ_{tot} was employed for all reactions considered.

As it can be appreciated from the simulations, decreasing the value of λ_{tot} results in an increased lifetime of charge recombination (slower reactions). For values of $\lambda_{tot} < 0.9$ eV the mean simulated recombination lifetimes is more than 1 second.

Section S4. Alternative electron transfer parameters within the "large driving force" scenario for A_1^- oxidation

In order to explore the impact of specific parameters in the simulations within the large driving force scenario, two further parameter sets were tested. In one case the free energies between the cofactors are those reported in the main text (also **Figure S7A**), but the values of $\lambda_{tot,F_X \to F_A}$ and $\lambda_{tot,F_A \to F_B}$ were changed to those employed the weak driving force

scenario, *i.e.* 0.7 eV and 0.9 eV, respectively. The latter value is also employed for all recombination rates, as done for the weak driving force case.

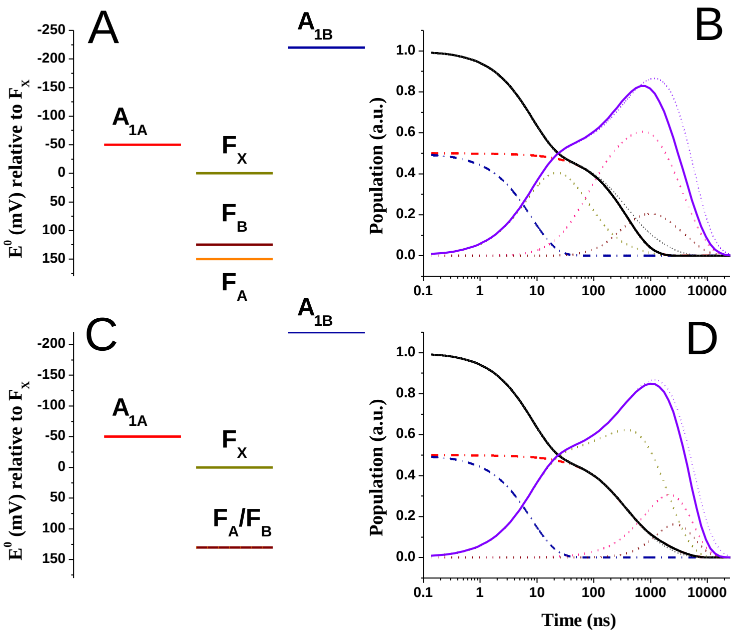

Figure S7. Panel A/C: energetic schemes for the large driving force A_1^- oxidation scenario as in the main text (**A**) and from Milanovsky *et al.* **[1]** (**C**). **Panel B:** Simulated ET kinetics, for energetics in Panel A and $\lambda_{tot,A_1\to F_X}$ = 1 eV, $\lambda_{tot,F_X\to F_A}$ = 0.7 eV and $\lambda_{tot,F_A\to F_B}$ = 0.9 eV. The latter value was used also for all recombination rates. **Panel D**: Simulated ET kinetics, for energetics in Panel C and λ_{tot} = 1 eV for all reactions considered. $A_{1A}^-(t)$: dashed-dot red line, $A_{1B}^-(t)$ dashed-dot blue line, $F_X^-(t)$ dotted golden line, $F_A^-(t)$ dotted orange line, $F_B^-(t)$ dotted burgundy line. Total population evolutions of $A_{1,tot}^-(t) = A_{1A}^-(t) + A_{1B}^-(t)$ (black line) and $FeS_{tot}^-(t) = F_X^-(t) + F_A^-(t) + F_B^-(t)$ (Violet). Dotted gray line and dotted violet lines are $A_{1,tot}^-(t)$ and $FeS_{tot}^-(t)$ from the main text "large driving force" simulations.

The results of the simulations are shown in **Figure S7B** where the comparison with the ET kinetics for $A_{1,tot}^-(t)$ and $FeS_{tot}^-(t)$ obtained for the parameters discussed in the main text are also presented. Concerning the $A_{1,tot}^-(t)$ oxidation the main difference resulting from the use of the different parameter sets is the suppression of the small amplitude long-lived phase, which gets negligible amplitude upon increasing the rate of ET between the FeS cluster, especially F_X^- oxidation, by lowering the reorganisation energy. The main, sub-microsecond phases of $A_{1,tot}^-(t)$ oxidation remains fundamentally unaltered. Concerning $FeS_{tot}^-(t)$ the main difference resides in an acceleration of the overall oxidation dynamics, which is a straightforward consequence of reducing the reorganisation energies. The total rise

in reduced $FeS_{tot}^{-}(t)$ population does not appear to be much affected. Nonetheless, the residence on each of the three clusters varies significantly, especially as a result of a more rapid F_X^- oxidation, characterised by a τ_{av} of 178 ns (similar to that obtained for the weak driving force model) with respect to ~1.5 μs, when an homogeneous value of λ_{tot} = 1 eV was employed. The predicted F_X^- oxidation are thus more in line with the experimental estimates for $\lambda_{tot,F_X \to F_A}$ = 0.7 eV. However, as already discussed in the main text the situation in which $\lambda_{tot,F_X \to F_A} < \lambda_{tot,A_1 \to F_X}$, although not impossible, appears unlikely. Moreover, even when considering $\lambda_{tot,F_X \to F_A}$ = 0.7 eV and $\lambda_{tot,F_A \to F_B}$ = 0.9 eV, the predicted lifetime for the recombination reaction is 980 ms (with respect to 573 ms for the homogenous λ_{tot} =1 eV simulations and 20-100 ms experimental values). Then, concerning the recombination reactions, the quality of the simulation worsen (**Figure S8**).

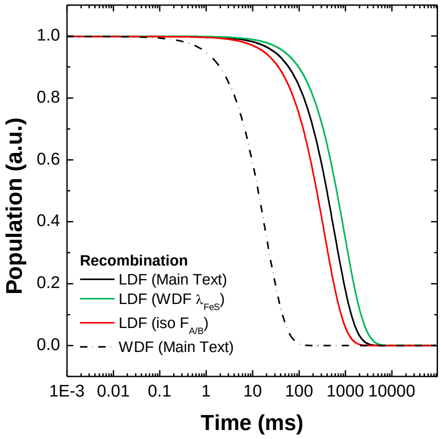

Figure S8. Comparison of the simulations of charge recombination reactions (in absence of an exit from the system (k_{out} =0) for the large driving force (LDF) scenario and parameters discussed in the main text (back solid line), and or the modification described in Figure S7, 1) energetics of Figure S7A and reorganisation energies for FeS-ET as in the weak driving force (WDF) model (solid green), 2) energetics of Figure S7C and λ_{tot} = 1 eV for all reactions considered (red). Dash-dotted lines are the simulation for the WDF as in the main text.

Simulations were also performed considering exactly the same value of ΔG^0 and E^0 reported by Milanovsky and coworkers **[39] (Figure S7C/D).** The main differences being that F_{A} and F_{B} are here considered as iso-potential with E^0 = – 500 mV (note they were considered collectively in the referred paper), and that $\Delta G^0_{F_X \to F_A}$ = – 130 meV (instead of 150 meV), so that $E^0_{F_X}$ = – 630 mV, 50 mV more positive than in the main text (and **Figure S7A**), and the same for both phylloquinones. An homogenous value λ_{tot} =1 eV was used for all ET

rates in the simulations, which are shown in **Figure 7D**. The ET kinetics for $A^-_{1,tot}(t)$ and $FeS^-_{tot}(t)$ obtained for the parameters discussed in the main text are also shown for comparison. It could be appreciated that the $A^-_{1,tot}(t)$ oxidation are basically unaffected, when decreasing $\Delta G^0_{F_X \rightarrow F_A}$. The main perturbation related to the predicted small-amplitude long-lived phase, which lifetime increased to 1.8 μs from 1.4 μs (the latter for the values used in the main text), whereas the amplitude decreased to 0.15 from 0.18. It is worth recalling this phase is not observed experimentally. The mean lifetime of F_X^- oxidation was simulated as 3.2 μs, slower than the 2..2 μs for the set of potentials used in the main text, and consistent with a decrease of 50 meV in the driving force. Again this values are about one order of magnitude slower than those estimated experimentally (~50-300 ns, and see **Figure S7B** for an experimentally compatible simulation of $F_X^-(t)$). The predicted lifetime for charge recombination reaction is 346 ms, faster than obtained for the main text parameter (573 ms) but still exceeding the experimental determination by a factor of ~3 (**Figure S8**).

Taken together it can be concluded that, decreasing the value of λ_{tot} to values similar to those used for the "weak" driving force scenario, does only slightly improves the match between experimental and simulated forward electron transfer kinetics involving the FeS cluster, but results in much less satisfactory simulation of both A_1^- oxidation and recombination reaction with P_{700}^+. Thus, the qualitative conclusion reported in the main text concerning ET between iron-sulphur clusters F_X, F_A and F_B within the "large driving force" scenario, are not strictly dependent on the specific value of λ_{tot} utilised which was chosen on the basis of simulating the better characterised A_1^- oxidation reaction.

Section S5. Further detail on the kinetic model employed to describe the redox cofactor population evolutions:

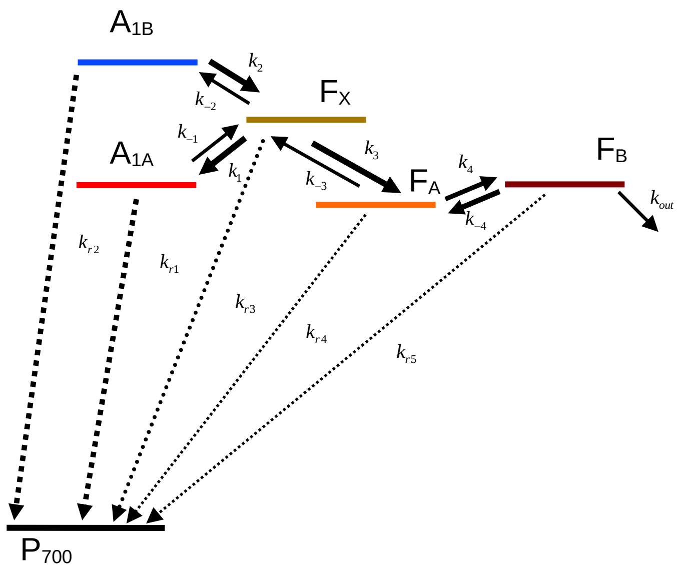

Figure S9. Kinetic scheme employed to calculate the PSI cofactor population evolutions. This is the same as in Figure 1B but all rates considered are indicated explicitly The solid arrows represent the forward and backwards rates between pairs of successive redox-active cofactors. The dashed ones represent charge recombination between the phyllo(semi)quinones and P_{700}^{+} whereas the dotted arrows indicate recombination between the reduced iron-sulphur clusters and P_{700}^{+}. The colour code is the same used throughout the text for the population evolution simulations. The energy levels are not on scale.

The population evolution of the redox cofactors, according to the kinetic scheme shown above (Figure S9), are obtained from the solution of a system of ordinary differential equations. The system, that can be expressed in compact matrix form as $\dot{\mathbf{P}}(t) = \mathbf{K}_i \cdot \mathbf{P}(t)$, is also given in the explicit form by the following set of linear differential equations:

$$\begin{cases} \dot{A}_{1A}^{-}(t) = -(k_1 + k_{r1})A_{1A}^{-}(t) + k_{-1}F_X^{-}(t) \\ \dot{A}_{1B}^{-}(t) = -(k_2 + k_{r2})A_{1B}^{-}(t) + k_{-2}F_X^{-}(t) \\ \dot{F}_X^{-}(t) = -(k_3 + k_{-1} + k_{-2} + k_{r3})F_X^{-}(t) + k_1 A_{1A}^{-}(t) + k_2 A_{1B}^{-}(t) + k_{-3}F_A^{-}(t) \\ \dot{F}_A^{-}(t) = -(k_4 + k_{-3} + k_{r4})F_A^{-}(t) + k_3 F_X^{-}(t) + k_{-4}F_B^{-}(t) \\ \dot{F}_B^{-}(t) = -(k_{out} + k_{-4} + k_{r5})F_B^{-}(t) + k_4 F_A^{-}(t) \end{cases} \qquad \text{Eqn. S1}$$

The (column) vector of the population evolution $\mathbf{P}(t)$ has then as its elements $(A_{1A}^{-}(t), A_{1B}^{-}(t), \ldots, F_B^{-}(t))$ and its first derivative with respect to time, $\dot{\mathbf{P}}(t) = (\dot{A}_{1A}^{-}(t), \dot{A}_{1B}^{-}(t), \ldots, \dot{F}_B^{-}(t))$. The rate matrix $\mathbf{K}_i$ is defined as:

$$\mathbf{K}_i = \begin{bmatrix} -(k_1+k_{r1}) & & k_{-1} & & \\ & -(k_2+k_{r2}) & k_{-2} & & \\ k_1 & k_2 & -(k_3+k_{-1}+k_{-3}+k_{r3}) & k_{-3} & \\ & & k_3 & -(k_4+k_{-3}+k_{r4}) & k_{-4} \\ & & & k_4 & -(k_{out}+k_{-4}+k_{r5}) \end{bmatrix}$$

The pair-wise rate constants k_i / k_{-i} represent forward and backward rates between each pair of cofactors (as indicated in the explicit system of ordinary differential equations, and the kinetic scheme in **Figure S9**) whereas k_{ri} describes the recombination between each cofactor and P_{700}^{+}.

The general solutions of the system of differential equation are, in vector form:

$$\mathbf{P}(t) = c_1\mathbf{V}_1 e^{\zeta_1 t} + c_2\mathbf{V}_2 e^{\zeta_2 t} + .. + c_i\mathbf{V}_i e^{\zeta_i t} \qquad \text{Eqn. S2}$$

where ζ_i are the eigenvalues of $\mathbf{K}_i$, (given by $\det(\mathbf{K}_i - \zeta\mathbf{I}_i) = 0$, where $\mathbf{I}_i$ is the identity matrix of order *i*), $\mathbf{V}_i$ are their corresponding eigenvectors ($\mathbf{K}_i\mathbf{V}_i = \zeta_i\mathbf{V}_i$) and c_j are scalars. It is then apparent that not only the eigenvalues, ζ_i, which relate to the experimental lifetimes by the relation $\tau_1 = -\zeta_i^{-1}$, but also the eigenvectors, $\mathbf{V}_i$, depend on all the actual *rate constants*, which is often neglected in the analysis of experimental values, perhaps for their complex relation.

Equation S2, the solution of the system of linear differential equations, can be put into explicit form as:

$$\begin{cases} A_{1A}^{-}(t) = c_1 v_{1,1} \exp^{\xi_1 t} + c_2 v_{2,1} \exp^{\xi_2 t} + c_3 v_{3,1} \exp^{\xi_3 t} + c_4 v_{4,1} \exp^{\xi_4 t} + c_5 v_{5,1} \exp^{\xi_5 t} \\ A_{1B}^{-}(t) = c_1 v_{1,2} \exp^{\xi_1 t} + c_2 v_{2,2} \exp^{\xi_2 t} + c_3 v_{3,2} \exp^{\xi_3 t} + c_4 v_{4,2} \exp^{\xi_4 t} + c_5 v_{5,2} \exp^{\xi_5 t} \\ F_{X}^{-}(t) = c_1 v_{1,3} \exp^{\xi_1 t} + c_2 v_{2,3} \exp^{\xi_2 t} + c_3 v_{3,3} \exp^{\xi_3 t} + c_4 v_{4,3} \exp^{\xi_4 t} + c_5 v_{5,3} \exp^{\xi_5 t} \\ F_{A}^{-}(t) = c_1 v_{1,4} \exp^{\xi_1 t} + c_2 v_{2,4} \exp^{\xi_2 t} + c_3 v_{3,4} \exp^{\xi_3 t} + c_4 v_{4,4} \exp^{\xi_4 t} + c_5 v_{5,4} \exp^{\xi_5 t} \\ F_{B}^{-}(t) = c_1 v_{1,5} \exp^{\xi_1 t} + c_2 v_{2,5} \exp^{\xi_2 t} + c_3 v_{3,5} \exp^{\xi_3 t} + c_4 v_{4,5} \exp^{\xi_4 t} + c_5 v_{5,5} \exp^{\xi_5 t} \end{cases} \qquad \text{Eqn. S3}$$

Where $v_{i,j}$ are the *j*-th elements of the *i*-th eigenvectors, $\mathbf{V}_i$. It is clear from the explicit form that the evolution of each cofactor considered is given by a linear combination of exponential functions, and that the number of exponentials phases is the same for all cofactors and it is equal to the number of states considered. Moreover, the value of each

exponential factor is the same for all cofactors/states considered. It is worth noting that this is the mathematical rationale behind the now very commonly employed "global" exponential fit analysis.

Equation S2 and S3 are general solutions, which describe the system evolution from any possible initial state and are therefore not univocal. The temporal evolution of each of the states considered needs then to be determined from a specific set of boundary conditions. Here, as rather customary, they have been chosen as the conditions approaching the initial excitation of the system ($t \to 0$), approximating the population of the phyllosemiquinones as instantaneous, since it occurs in the tens of picoseconds timescale which is several orders of magnitude faster than the population evolutions of interest (that are in the nanosecond to microsecond time window instead). Solving Equations S2 (or the explicit form Eqn. S3) for the initial conditions, that in this case were chosen as $A_{1A}^{-}(0)$ =0.5, $A_{1B}^{-}(0)$ = 0.5, $F_X(0)$ =0, $F_A(0)$ =0, $F_B(0)$ = 0 returns the scalar coefficients c_i . The amplitude factors, p_i, reported throughout the text are then the products $c_i v_{i,j}$ that can be compared to the experimentally retrieved pre-exponential amplitudes.
